# Electroacupuncture improves TBI dysfunction by targeting HDAC overexpression and BDNF-associated Akt/GSK-3β signaling

**DOI:** 10.3389/fncel.2022.880267

**Published:** 2022-08-09

**Authors:** Shih-Ya Hung, Hsin-Yi Chung, Sih-Ting Luo, Yu-Ting Chu, Yu-Hsin Chen, Iona J. MacDonald, Szu-Yu Chien, Peddanna Kotha, Liang-Yo Yang, Ling-Ling Hwang, Nae J. Dun, De-Maw Chuang, Yi-Hung Chen

**Affiliations:** ^1^Graduate Institute of Acupuncture Science, China Medical University, Taichung, Taiwan; ^2^Division of Colorectal Surgery, China Medical University Hospital, Taichung, Taiwan; ^3^Graduate Institute of Medical Sciences, College of Medicine, Taipei Medical University, Taipei, Taiwan; ^4^Department of Physiology, School of Medicine, College of Medicine, China Medical University, Taichung, Taiwan; ^5^Laboratory for Neural Repair, China Medical University Hospital, Taichung, Taiwan; ^6^Department of Pharmacology, Temple University School of Medicine, Philadelphia, PA, United States; ^7^Intramural Research Program, National Institute of Mental Health, National Institutes of Health, Bethesda, MD, United States; ^8^Chinese Medicine Research Center, China Medical University, Taichung, Taiwan; ^9^Department of Photonics and Communication Engineering, Asia University, Taichung, Taiwan

**Keywords:** electroacupuncture, preclinical traumatic brain injury, motor function tests, histone deacetylases, BDNF, Akt/GSK-3β

## Abstract

**Background:**

Acupuncture or electroacupuncture (EA) appears to be a potential treatment in acute clinical traumatic brain injury (TBI); however, it remains uncertain whether acupuncture affects post-TBI histone deacetylase (HDAC) expression or impacts other biochemical/neurobiological events.

**Materials and methods:**

We used behavioral testing, Western blot, and immunohistochemistry analysis to evaluate the cellular and molecular effects of EA at LI4 and LI11 in both weight drop-impact acceleration (WD)- and controlled cortical impact (CCI)-induced TBI models.

**Results:**

Both WD- and CCI-induced TBI caused behavioral dysfunction, increased cortical levels of HDAC1 and HDAC3 isoforms, activated microglia and astrocytes, and decreased cortical levels of BDNF as well as its downstream mediators phosphorylated-Akt and phosphorylated-GSK-3β. Application of EA reversed motor, sensorimotor, and learning/memory deficits. EA also restored overexpression of HDAC1 and HDAC3, and recovered downregulation of BDNF-associated signaling in the cortex of TBI mice.

**Conclusion:**

The results strongly suggest that acupuncture has multiple benefits against TBI-associated adverse behavioral and biochemical effects and that the underlying mechanisms are likely mediated by targeting HDAC overexpression and aberrant BDNF-associated Akt/GSK-3 signaling.

## Introduction

Traumatic brain injury (TBI) imposes enormous economic and health burdens upon affected individuals, their families, and society as a whole (Humphreys et al., 2013). TBI is described as a chronic disease process with ongoing effects on multiple organ systems (Masel and DeWitt, 2010) as well as psychological distress that is associated with changes in mood, cognition, and other behaviors affecting the daily life of TBI survivors (Kreuter et al., 1998; Rabinowitz and Levin, 2014). Symptoms resulting from the injury may be apparent immediately or only appear days or even weeks afterwards (Masel and DeWitt, 2010). Mild TBI may cause only temporary confusion and headache, while moderate and severe TBI are associated with more disabling neurological and functional impairments (Obermann et al., 2015; Capizzi et al., 2020); TBI associated with the greatest clinical severity can lead to unconsciousness, amnesia, coma, and even death (McKee and Daneshvar, 2015). Currently, there are some neurosurgical treatment options for TBI, such as decompressive craniotomy and specialized neurointensive care for TBI victims has been reported to reduce the mortality of severely affected patients (Bentz et al., 2010; Rosenfeld et al., 2012). However, no pharmaceutical treatments have proven clinically effective in TBI, and the U.S. Food and Drug Administration has approved no treatments or interventions for this brain trauma (Wang et al., 2021; Tabet et al., 2022).

In TBI, primary injury due to axonal shearing and hemorrhage of brain tissue is followed by secondary injury involving excitotoxicity, cortical spreading depression, ischemia, mitochondrial dysfunction, apoptosis, necrosis, and inflammation (Bentz et al., 2010; Hartings et al., 2011; Karve et al., 2016; Nakamura et al., 2020). Neuroinflammation is a major event in the secondary response to TBI, marked by activation of astrocytes and microglia and the release of proinflammatory cytokines from these activated cells into the microenvironment (Block and Hong, 2005; Chio et al., 2010; Karve et al., 2016). Increasing evidence suggests that histone deacetylase (HDAC) activity is altered after central nervous system (CNS) injury and that HDAC inhibition suppress the inflammatory response in activated microglia (Suh et al., 2010; Kannan et al., 2013). Pharmacological inhibition of HDACs has also been shown to be neuroprotective by suppressing inflammatory responses and neuronal loss as well as promoting neuronal rewiring after CNS injury (Zhang et al., 2018). Moreover, TBI-associated physiological and cognitive deficits are attenuated by upregulation in levels of brain-derived neurotrophic factor (BDNF) and inhibition of glycogen synthase kinase-3 (GSK-3) activity via activation of upstream Akt (Zhao et al., 2012; Leeds et al., 2014).

Brain-derived neurotrophic factor is a critically important neurotrophin in the development, plasticity, neurogenesis, and neuroprotection of the CNS (Autry and Monteggia, 2012). After binding to its receptor tropomyosin receptor kinase B (TrkB), BDNF activates phosphatidylinositol 3-kinase (PI3K), phosphorylating and activating serine/threonine kinase Akt, a major cell survival factor that in turn phosphorylates and inactivates GSK-3, an evolutionally conserved kinase comprising α and β isoforms (Kaidanovich-Beilin and Woodgett, 2011; Leeds et al., 2014). After TBI, activation of GSK-3 constitutively inhibits neuroprotective processes and promotes the expression of proapoptotic proteins (Leeds et al., 2014; Shim and Stutzmann, 2016). GSK-3 inhibition has been proposed as a promising target in the development of new therapies for TBI and related brain insults via mechanisms primarily involving changes in gene expression (Chuang et al., 2011; Leeds et al., 2014; Shim and Stutzmann, 2016).

No effective pharmacological therapies are yet available for managing post-TBI neurodegenerative processes at present (Morales et al., 2005; Leeds et al., 2014; Crupi et al., 2020). In this context, manual acupuncture and electroacupuncture (EA) have both been found to be neuroprotective in rodent models of Parkinson’s disease (Ko et al., 2019) and vascular dementia (Yang et al., 2018). Furthermore, acupuncture was found to facilitate neural repair in experimental TBI by inducing the proliferation and differentiation of endogenous neural stem cells in injured brain tissue (Nam et al., 2013; Jiang et al., 2016), and EA was found to significantly attenuate TBI-associated brain microgliosis and astrogliosis, as well as neuronal apoptosis; Although BDNF has been suggested to play a role in these processes (Tang et al., 2016), the detailed underlying mechanisms have not yet been elucidated. Clinical studies also suggest that acupuncture or EA are effective and safe in the acute management and rehabilitation of TBI (Wong et al., 2012). However, it remains unclear whether acupuncture normalizes TBI-induced neurological complications or regulates HDAC expression post-TBI, which in turn impacts other biochemical/neurobiological events.

To explore these issues, this study examined the cortical tissue from the TBI mice of weight drop-impact acceleration (WD) model or a controlled cortical impact (CCI) model, both of which mimic the clinical consequences of injury mechanisms in human TBI. The acute and long-term neuroprotective properties and anti-neuroinflammatory effects of EA treatment were investigated, with a focus on the roles of HDAC isoforms and BDNF-associated Akt/GSK-3 signaling in TBI symptomatology.

## Materials and methods

### Experimental animals and research ethics

All experiments were performed in strict accordance with the guidelines and approval of the Institutional Animal Care and Use Committee of China Medical University, Taiwan (approval number: CMUIACUC-2019-075-1). C57BL/6 mice (9-11 weeks; BioLasco Taiwan Co., Ltd., Taipei, Taiwan) used in these experiments had free access to food and water and were housed in an animal research facility that controlled for relative humidity and temperature under a 12:12 h light/dark cycle. The number of animals used in each test followed the instruction of statistical analysis in a previous study (Arifin and Zahiruddin, 2017).

### Weight drop-impact acceleration-induced traumatic brain injury

Each mouse underwent an anesthetic protocol using an intraperitoneal (i.p.) injection of solutions containing tiletamine-zolazepam (50 mg/kg; Zoletil, Vibac, France) and xylazine (10 mg/kg; Rompun, Bayer, Leverkusen, Germany) 15 min prior to TBI. Mice in the Control group underwent the anesthesia protocol but without TBI. TBI was induced using the modified Marmarou closed head impact acceleration model (Marmarou et al., 1994); the mouse was placed on its abdomen, in a prone position, on flexible foam under a device consisting of a Plexiglas tube positioned vertically over the animal’s head. TBI was induced using a 50 g metal weight falling from a height of 80 cm through a vertical stainless tube (1.8 cm circumference), striking the skull (Supplementary Figure 1).

### Controlled cortical impact-induced traumatic brain injury

Traumatic brain injury was induced using a CCI device (Precision Systems and Instrumentation, LLC, Fairfax, VA, United States), as previously described (Lin et al., 2014). Briefly, each animal underwent an anesthetic protocol using an i.p. injection of tiletamine-zolazepam (50 mg/kg; Zoletil, Vibac, France) combined with xylazine (10 mg/kg; Rompun, Bayer, Leverkusen, Germany) 15 minutes before TBI impact, for which they were placed in a stereotaxic frame with an adaptor (Kopf Instruments, Tujunga, CA, United States). Mice in the Control group were traded with the anesthetic protocol but without TBI. A 5-mm-diameter craniotomy was performed over the left parietal cortex between the bregma and the lambda, 1 mm lateral to the midline. The point of impact was identified midway between the lambda and bregma sutures, as well as midway between the central suture and the left temporalis muscle. CCI injury was performed using a 3-mm-diameter convex tip set to compress the brain by 1.5 mm, at a speed of 5.0 m/s and a depth of 2 mm for 500 ms dwell time, as previously described (Yu et al., 2012). The bone flap was then replaced. Body temperature was maintained at 37 ± 0.5°C with a heating pad coupled to a rectal probe.

### Electroacupuncture treatment

**FIGURE 1:**
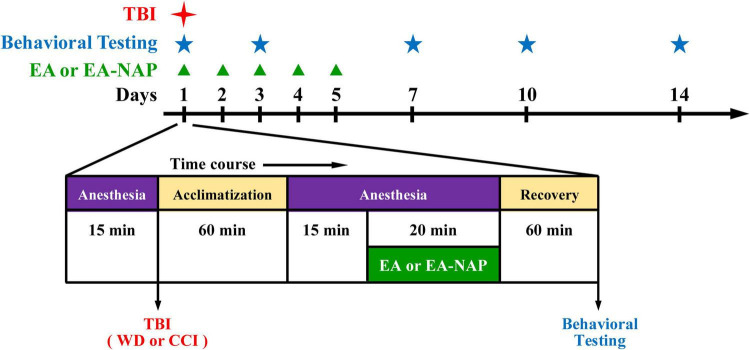
Timeline for the study procedures including traumatic brain injury (TBI), anesthesia, electroacupuncture (EA), EA at non-acupoints (EA-NAP), recovery, and behavioral testing.

Electroacupuncture procedures followed those published in previous reports (Chen et al., 2013; Lin et al., 2016). Briefly, mice were individually acclimated in rectangular observation boxes for 1 h, then anesthetized with 1.5% isoflurane for 15 min. Under anesthesia, a pair of stainless-steel acupuncture needles were inserted 2 mm deep into the murine equivalents of the human LI4 and LI11 acupoints. The proportional locations of the LI4 and LI11 acupoints in the mouse were determined using anatomical descriptions in the World Health Organization guidelines for human acupoints (World Health Organization, 2008). In mice, LI4 is located on the first dorsal interossei, radial to the midpoint of the second metacarpal bone in the forelimb; LI11 is located in the depression on the lateral end of the cubital crease in the forelimb when the elbow is fully flexed (Supplementary Figure 2). For mice with EA at non-acupoints (EA-NAP), needles were inserted bilaterally into the middle of the lateral deltoid muscle, a nonmedian nerve-innervated location (as the sham acupuncture). EA stimuli were delivered by an EA Trio 300 stimulator (Ito, Japan) at 2-mA intensities for 20 min at 2 Hz, with a pulse width of 150 μs (Han et al., 2008). After each EA or EA-NAP treatment session, the mice were allowed to recover for 1 h before undergoing behavioral testing. EA or EA-NAP treatment was initially administered 1 h after either WD-TBI or CCI-TBI on Day 1, then again on Days 2, 3, 4, and 5 (Figure 1).

### Rotarod testing

The rotarod test is a sensitive method for measuring sensorimotor function and motor coordination (Fujimoto et al., 2004; Gold et al., 2013) and is commonly used to evaluate the neurological effects of drugs or trauma on the motor coordination of rodents (Onyszchuk et al., 2007). This method has also been applied to evaluate the motor deficits in the TBI models (Pearl et al., 1969; Hamm et al., 1994; Elder et al., 2012). For the rotarod protocol, mice were placed on a rotarod cylinder (Ugo Basile S.R.L., Gemonio, Italy) with an accelerating protocol. Briefly, the speed was slowly increased from 4 to 40 revolutions per minute (RPM), using an acceleration rate of 5 RPM/10 s. Latency to fall from the device or to cling and rotate was recorded for three full rotations. Test data are presented as the average of the three recorded times that a mouse remained on the rotarod compared with the internal baseline (before WD-TBI). Rotarod testing was conducted on Days 1, 3, 7, and 14 post-TBI based on previous studies (Namjoshi et al., 2017; Zhong et al., 2017; Gong et al., 2022; Tang et al., 2022).

### Motor coordination and balance test (beam walk test)

Performance on this test is believed to reflect functional changes in affected brain regions, i.e., the motor cortex, sensory cortex, thalamus, brainstem, and cerebellum (Hallam et al., 2004; Lee et al., 2004). Briefly, assessments of motor coordination and balance were conducted on Days 1, 3, and 7 post-TBI with a beam walk device, as previously described (Yu et al., 2012). The apparatus consisted of a 1-meter beam with a flat surface of 6 mm in width and 12 mm in circumference, suspended 50 cm above the benchtop on two poles. The time to complete the beam walk and the number of foot faults were counted over 80 cm of travel. Sawdust and commercial pellets were placed in the box. On the training day only, the mice were pushed by hand to encourage them to run along the beam to their home box. The day before testing, the mice were trained to cross the beam within 10 s. Each testing session commenced with 1 h of acclimatization. After each use, the apparatus was cleaned with tissue paper soaked in 75% alcohol.

### Adhesive removal test

The test distinguishes motor functions and sensorimotor deficits caused by unilateral lesions placed in distinct areas of the rat somatic sensorimotor cortex (the caudal forelimb region, rostral forelimb region, or anteromedial cortex) (Barth et al., 1990; Fleming et al., 2013; Li et al., 2018). This measure is also the only test with sufficient sensitivity to reveal long-term deficits caused by relatively small brain lesions (Bouet et al., 2009). Briefly, on Days 1, 3, and 7, an adhesive sticker (cut into equal-sized squares, 4 mm in diameter) was applied to the hair-free areas (the thenar, hypothenar, and interdigital pads) of the right forepaw (Bouet et al., 2009). The mice were placed back into the test cage, and the times taken to find the sticker and remove it were recorded for a maximum of 120 s. In each trial, an adhesive trial was applied twice to the right forepaw. Each trial set consisted of two trials that were averaged and analyzed for each animal.

### Y-maze test

Ten days after CCI-TBI, mice were tested in a white plastic maze apparatus consisting of three enclosed arms, each 50 cm long, 11 cm wide, and 10 cm high, set at an angle of 120° to each other, in the shape of a Y (Yau et al., 2007). Testing involved two trials, separated by a two-hour interval. In the first (acquisition) trial, one arm (the novel arm) was blocked. After the mice were placed at the end of a pseudo-randomly chosen arm (the start arm), they were allowed to explore the maze for 5 min before being returned to their home cages for 2 h. In the second (retention) trial, mice were allowed to explore all three arms for 5 min. The time spent exploring each arm was video-recorded by an observer blinded to the treatment condition. The percentage of time spent in the novel arm vs. the total time spent in all three arms during the first 5 min of the retention trial was analyzed, as previously described (Yau et al., 2007).

### Western blot analysis

All groups underwent isoflurane anesthesia, as previously described (Lin et al., 2016). The entire cortex was sampled from the WD-TBI mice for Western blot analysis, whereas only the injured cortex was sampled from the CCI-TBI mice. The tissue was homogenized in tissue lysis buffer containing protease inhibitors and phosphatase inhibitors. Each sample of protein concentration was evaluated using a Pierce BCA Protein Assay Kit. Proteins (∼25 μg) were resolved by 8–12% SDS-PAGE under reducing conditions, and the gel was transferred to PVDF membranes (Pall Corporation, Port Washington, New York, NY, United States), then incubated overnight at 4°C with primary antibodies for HDAC1 (#5356; Cell Signaling Technology, Danvers, MA, United States), HDAC2 (#5113; Cell Signaling Technology), HDAC3 (#3949; Cell Signaling Technology), ionized calcium-binding adapter molecule 1 (Iba1) (ab5076; Abcam, Cambridge, United Kingdom), glial fibrillary acidic protein (GFAP) (#3670; Cell Signaling Technology), tumor necrosis factor-alpha (TNF-α) (ab9739; Abcam), phosphorylated-GSK-3β Ser9 (p-GSK-3β) (GTX132997; GeneTex, Irvine, CA, United States), GSK-3β (GTX133372; GeneTex), phosphorylated-Akt Ser473 (p-Akt) (GTX128414; GeneTex), Akt (GTX121937; GeneTex), BDNF (ab108319; Abcam), or Bax (#2772; Cell Signaling Technology). Using β-Actin antibody (GTX629630; GeneTex) as a loading control. Membranes were incubated with secondary antibodies (anti-rabbit IgG-HRP sc-2004; anti-mouse IgG-HRP sc-2005; or anti-goat IgG-HRP sc-2020; Santa Cruz, Dallas, TX, United States). Protein bands were detected and estimated by an enhanced chemiluminescence reagent (ECL) (Millipore, Billerica, MA, United States) using an ImageQuant LAS 4000 camera (GE Healthcare, Little Chalfont, United Kingdom), and densitometry was performed using Fusion-Capt Software (Labtech International, Inc., Vilber Lourmat, France).

### RNA extraction, reverse-transcription, and real-time quantitative polymerase chain reaction

Total RNAs were extracted from cortical tissues of mice by using Trizol (Thermo Fisher Scientific, Waltham, MA, United States). Single-strand cDNAs were synthesized using a high-capacity cDNA Reverse Transcription kit (Thermo Fisher Scientific). Real-time quantitative PCR was performed to analyze *BDNF*, *TNF-α*, *interleukin* (*IL*)-*6*, and *β-actin* mRNA levels by the StepOne Plus real-time PCR system (Thermo Fisher Scientific). TaqMan™ Gene Expression Assays (Thermo Fisher Scientific) of *TNF-α* and *IL-6* were used as the primer and probe set for *TNF-α* and *IL-6* in real-time quantitative PCR reactions. The forward (F) and reverse (R) primer and probe (P) sequences for mouse *BDNF* were 5′-CCGAGAGCTTTGTGTGGAC-3′ (F), 5′-TCATGCAACCGAAGTATGAAA-3′ (R), and TTCCACCA (P). The primer and probe sequences for mouse *β-actin* were 5′-ACTGCTCTGGCTCCTAGCAC-3′ (F), 5′-CCACCGATCCACACAGAGTA-3′ (R), and CTCCTCCT (P). Thermal cycling conditions of real-time quantitative PCR were 95°C for 10 min, 40 cycles at 95°C for 10 s, 55°C for 30 s, and then 72°C for 10 s. *BDNF*, *TNF-α*, and *IL-6* mRNA expression levels were normalized with *β-actin* as the control and then expressed as folds of control by the ΔΔCt method.

### Immunohistochemistry staining

The experimental procedures were conducted as previously described (Lin et al., 2016). Briefly, mice were anesthetized with urethane (1.2 g/kg; i.p.) and perfused intracardially with chilled 0.1 M PBS. Brains were removed and fixed in 10% formalin solution for 2 days at 4°C. Tissue samples were transferred to 30% sucrose/PBS solution for at least 1 day before sectioning. Coronal-30-μm-thick brain sections were prepared at –25°C using standard frozen section procedures. Brain sections from each mouse were randomly selected for IHC evaluations. Brain sections were treated with 3% hydrogen peroxide to eliminate endogenous peroxidase activity. The nonspecific binding sites in sections were blocked with bovine serum albumin and incubated overnight with an antibody against Iba1 (019-19741; Wako Chemicals USA, Richmond, VA, United States) and an antibody against GFAP (018-27283; Wako Chemicals USA). Sections were incubated for 1 h with biotinylated secondary antibody, then for 30 min with avidin-biotin-peroxidase complex (ABC kit, Vector Laboratories). Finally, labeling was visualized by incubation with 0.01% hydrogen peroxide and 0.05% 3,3’-diaminobenzidine. Sections were mounted on slides with 0.25% gel alcohol, air-dried, dehydrated with graded ethanol (50, 70, and 95% for 6 min each), followed by incubation with 100% ethanol for 10 min and xylene (three times for 10 min each). The stained sections were scanned with a NanoZoomer-XR digital slide scanner (Hamamatsu Photonics K.K., Hamamatsu, Japan) and imaged with its NDP View2 software.

### Measurement of histone deacetylase 3 activity

Forty-eight hours after CCI, cortical tissue was dissected from the ipsilateral side for nuclear protein separation using a cytoplasmic and nuclear protein extraction kit (BRARZ106; TOOLs, Taiwan) and the concentration of each nuclear protein was determined by the Pierce™ BCA Protein Assay Kit (23225; ThermoFisher Scientific, CA, United States). HDAC3 enzymatic activity of each nuclear protein was measured using the HDAC3 Activity Fluorometric Assay Kit (K343-100; BioVision, CA, United States). All procedures were conducted according to the manufacturer’s instructions. Briefly, 4–6 μg nuclear protein of each sample was diluted in HDAC3 Assay Buffer and incubated for 30 min at 37°C with HDAC3 substrate. After five minutes of development at 37°C, the fluorescence intensity of HDAC3 activity was measured using a microplate reader (Bio-Tek Synergy HT, VT, United States) with excitation at 380 nm and emission at 500 nm. Following the manufacturer’s instructions, trichostatin A and HDAC3 protein were used as the background and positive control, respectively. The HDAC3 activity of each sample is expressed as the relative fluorescence level of the CCI-TBI sample (percentage of CCI group).

### Statistical analysis

All data analyses were performed using GraphPad Prism 5 software (GraphPad Software, Inc., La Jolla, CA, United States). Quantitative data are expressed as the mean ± standard error of the mean (S.E.M.). Significant differences between the two independent groups were determined via Student’s *t*-test. For three or more independent groups, one-way ANOVA with Newman-Keuls multiple comparison testing was used to compare differences. A *p*-value of < 0.05 was considered to be statistically significant.

## Results

In the behavioral tests, TBI was followed by five days of once-daily EA treatment administered at specific acupoints (LI4 and LI11) that are believed to facilitate recovery from TBI, or at locations that are deemed to have no such effects (non-acupoints) (Wong et al., 2012; Wolf et al., 2015; Cavalli et al., 2018). On Days 1, 3, 7, 10, and 14, the mice underwent behavioral testing. A flowchart of the study timeline is shown in Figure 1.

### Electroacupuncture at LI1 and L14 acupoints improved functional outcomes in weight drop-impact acceleration-induced traumatic brain injury mice

**FIGURE 2:**
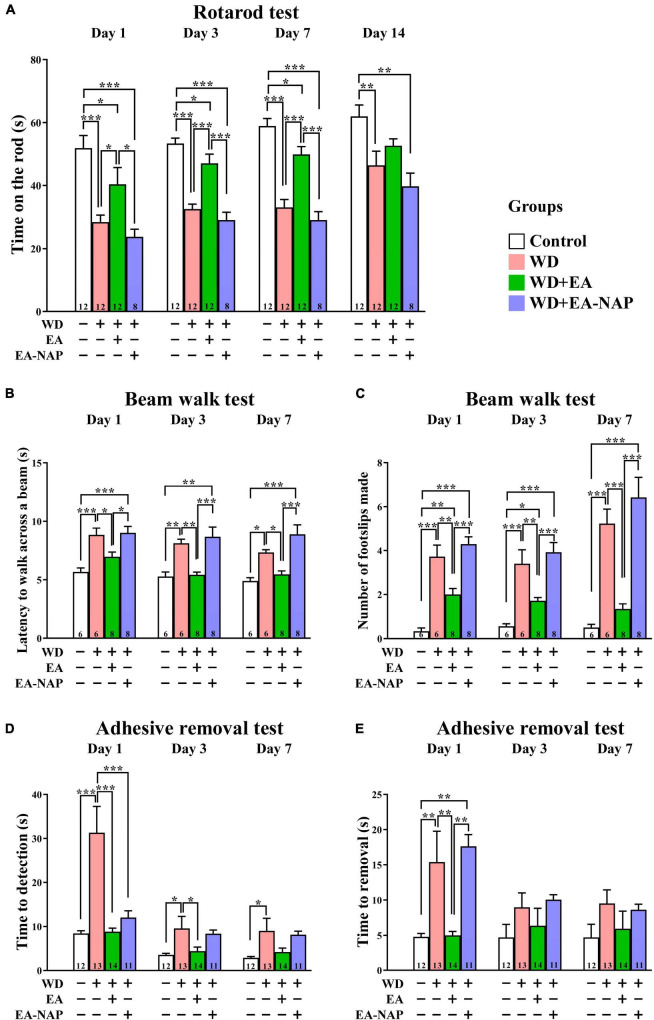
EA treatment markedly improved multiple functional outcomes after WD-TBI. **(A)** Accelerating rotarod test, **(B,C)** balance and motor coordination testing (beam walk test), **(D,E)** adhesive removal test. In all behavioral tests, mice in WD group displayed severe functional deficits compared to the Control group. WD+EA group significantly attenuated those deficits. WD+EA-NAP group had no significant effect on any deficits at any time of testing, except on Day 1 in **(D)**. Data are the means ± S.E.M. of at least three independent experiments. *p < 0.05, **p < 0.01, ***p < 0.001 (statistical testing was performed by one-way ANOVA/Newman-Keuls test). The number underneath each bar in **(A–E)** refers to the number of mice used in the study group. The detailed data points are presented in Supplementary Figure 3. The *F* values of one-way ANOVA for **(A–E)** are presented in Supplementary Table 1.

To test the hypothesis that EA treatment would improve motor and sensory function in TBI mice, the effect of WD-TBI on functional outcomes was evaluated by the accelerating rotarod, beam walk, and adhesive removal tests (see Figure 2). Latency to fall from the accelerating rotarod was markedly shortened by approximately 30-50% among mice in the WD group compared with the Control group on Days 1, 3, 7, and 14 (*p <* 0.001, *p <* 0.001, *p <* 0.001, and *p <* 0.01, respectively, Figure 2A). WD-induced endurance deficits in the rotarod test were significantly reversed by post-TBI treatment with EA (WD+EA group) on Days 1, 3, and 7 (*p <* 0.05, *p <* 0.001 and *p <* 0.001, respectively). In contrast, rotarod performance did not significantly differ on any of the testing days between the WD+EA-NAP group and WD group.

The beam walk test was used to evaluate the effects of EA on motor coordination in WD-TBI mice. The time taken to complete the beam walk revealed that the WD and the WD+EA-NAP group were both significantly impaired on post-TBI Days 1 and 3 compared with the Control group (*p <* 0.01 and *p <* 0.05, respectively, Figure 2B). Interestingly, only the WD+EA-NAP group remained significantly more impaired than the Control group on Day 7 (*p <* 0.001), suggesting that the WD group recovered faster than the WD+EA-NAP group. The performance of the WD+EA group was significantly improved on Days 1, 3, and 7 compared with the WD (*p <* 0.05, *p <* 0.01, and *p <* 0.05, respectively, Figure 2B) and WD+EA-NAP group (*p <* 0.05, *p <* 0.001, and *p <* 0.001, respectively, Figure 2B). In addition, the number of foot faults was significantly increased in the WD and WD+EA-NAP group on Days 1, 3, and 7 compared with the Control group (*p <* 0.001 in all cases), and significantly fewer foot faults occurred in WD+EA group compared with the WD group on Days 1, 3, and 7 (*p <* 0.01, *p <* 0.01, and *p <* 0.001, respectively, Figure 2C).

The adhesive removal test was used to understand how EA affects sensorimotor function in TBI mice. Here, the time taken to find the adhesive pad was significantly prolonged in the WD group compared with the Control group on Days 1, 3, and 7 (*p <* 0.001, *p <* 0.05, and *p <* 0.05, respectively, Figure 2D) with about a four-fold increase on Day 1. In the WD+EA group, the time taken to find the adhesive pad on Days 1 and 3 was significantly shortened compared to the WD group (*p <* 0.001 and *p <* 0.05, respectively); the between-group difference remained shorter in the WD+EA group on Day 7 but was no longer significant. In addition, the time taken to find the pad was significantly shorter in the WD+EA-NAP group compared with the WD group on Day 1, but no other significant between-group benefits were observed on any other testing days in the WD+EA-NAP group. Moreover, the time to remove each pad on Day 1 was prolonged three-fold in the WD group compared with the Control group (*p <* 0.01, Figure 2E). EA treatment blocked the WD-TBI-induced increase in time taken to remove the adhesive pad on Day 1 in the WD+EA group compared with the WD group (*p <* 0.01). Interestingly, the WD+EA-NAP group took a longer time to remove the adhesive pads on Day 1 compared with the Control group (*p <* 0.01) and the WD+EA group (*p <* 0.01). No significant between-group differences in adhesive removal times were observed on Days 3 and 7.

### Electroacupuncture treatment reduced levels of histone deacetylase 1 and histone deacetylase 3 in weight drop-impact acceleration-induced traumatic brain injury mice

**FIGURE 3:**
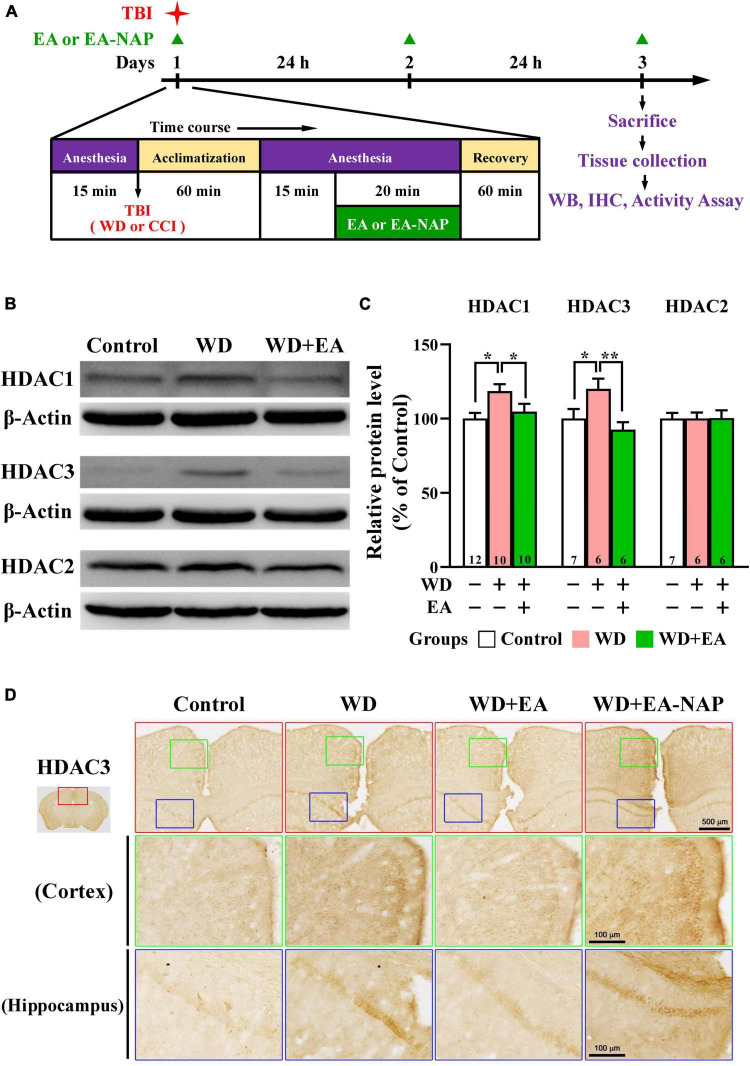
EA treatment reversed WD-TBI-induced elevations in HDAC1 and HDAC3 expression in murine cortex tissue. **(A)** Timeline for the study procedures including TBI, anesthesia, EA, EA-NAP, and recovery. Mice were sacrificed 48 h after brain injury. **(B)** Representative Western blot results show levels of HDAC1, HDAC3, and HDAC2 protein expression in murine cortex 48 h after WD-TBI. The brains were removed and the entire cortex was dissected for Western blotting analysis as described in the “Materials and methods.” β-Actin was used as the loading control. **(C)** Quantitative analysis of HDAC1/3/2 protein expression levels in **(B)**. **(D)** Representative images of IHC staining show anti-HDAC3 antibody immunoreactivity in the cortical and hippocampal areas of the Control, WD, WD+EA, and WD+EA-NAP groups. High magnification images from the top panels (red box) are shown in the green and blue boxes, respectively. Scale bars of red box panels are 500 μm; of the green and blue box panels are 100 μm. Data are the means ± S.E.M. of at least three independent experiments. *p < 0.05, **p < 0.01 (statistical testing was performed by one-way ANOVA/Newman-Keuls test). The number underneath each bar in **(C)** refers to the number of mice used in the study group. The original Western blot images are presented in Supplementary Figure 4. The number underneath each bar in **(C)** refers to the number of mice used in the study group. The *F* values of one-way ANOVA for **(C)** are presented in Supplementary Table 2.

Because the overexpression of certain HDAC isoforms is known to be detrimental to the nervous system, and because HDAC inhibitors in general exhibit neuroprotective effects (Chuang et al., 2009), the effects of TBI and EA on protein levels of HDAC isoforms in the mouse brain cortex were evaluated. Cortical levels of HDAC1, HDAC2, and HDAC3 were assessed by Western blotting 48 h post-WD injury. EA was performed once daily for three consecutive days (Figure 3A). Levels of HDAC1 and HDAC3 were significantly upregulated in the WD group compared with the Control group (*p* < 0.05 in all cases, Figures 3B,C). HDAC1 and HDAC3 levels were markedly downregulated in the WD+EA group compared with the WD group (*p* < 0.01 and *p* < 0.001, respectively, Figures 3B,C), suggesting that EA treatment inhibited HDAC1 and HDAC3 overexpression after WD-TBI. No significant between-group differences were observed in cortical HDAC2 levels between the Control, WD, and WD+EA groups (Figures 3B,C), suggesting that HDAC2 levels were unaffected by TBI or by EA treatment after WD-TBI. Similar results for HDAC3 expression were also shown in the IHC analysis (Figure 3D). The HDAC3 levels of mouse cortical and hippocampal areas were evaluated in the Control, WD, WD+EA, and WD+EA-NAP groups. Representative images in the Control group revealed scant, weakly-stained HDAC3-positive cell bodies. In contrast, cortex sections from the WD group showed numerous, densely situated, and strongly labeled HDAC3-positive cells; these features were strongly attenuated in the WD+EA group. Tissue from WD+EA-NAP mice exhibited strongly-stained HDAC3-positive cells, like those in the WD group tissue.

### Electroacupuncture treatment inhibited weight drop-impact acceleration-induced neuroinflammation

**FIGURE 4:**
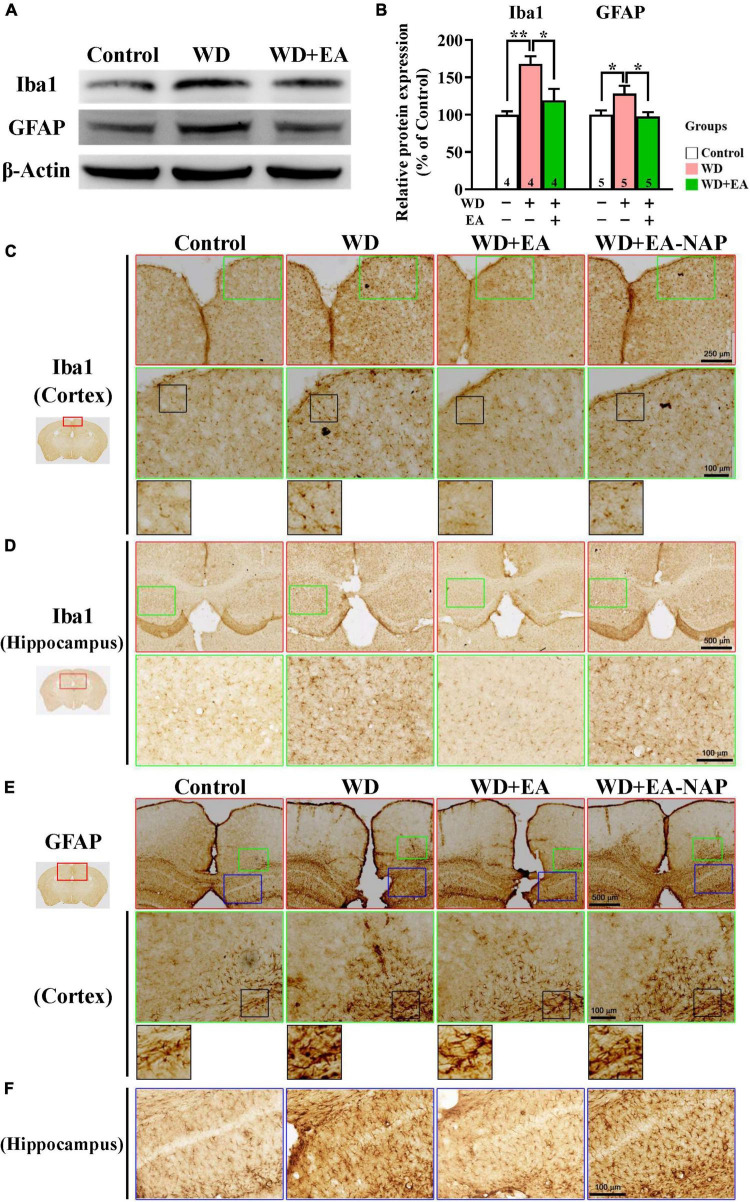
EA treatment attenuated WD-TBI-induced activation of microglia and astrocytes. **(A)** Representative Western blot results show levels of Iba1 and glial fibrillary acidic protein (GFAP) at 48 h in the mouse cortex from the Control, WD, and WD+EA groups. β-Actin was used as the loading control. **(B)** Quantitative analysis of Iba1 and GFAP protein expression levels in **(A)**. **(C)** Representative images of IHC staining show anti-Iba1 **(C,D)** and anti-GFAP **(E,F)** antibody immunoreactivity in cortical and hippocampal areas of the Control, WD, WD+EA, and WD+EA-NAP groups. High magnification images marked by the small green or blue boxes from the top panels (red box) are shown below. The bottom panels in **(C)** and **(E)** show higher-magnification images of the cortex from the middle panels marked by the small black boxes. Scale bars of the red box panels in **(C)** are 250 μm; of the red box panels in **(D)** are 500 μm; of the green box panels in **(C–E)** are 100 μm; of the blue box panels in **(F)** are 100 μm. Data are the means ± S.E.M. of at least three independent experiments. *p < 0.05, **p < 0.01 (statistical testing was performed by one-way ANOVA/Newman-Keuls test). The number underneath each bar in **(B)** refers to the number of mice used in the study group. The original Western blot images are presented in Supplementary Figure 5. The *F* values of one-way ANOVA for **(B)** are presented in Supplementary Table 3.

To study the effects of EA on WD-induced neuroinflammation, the activation of microglia and astrocytes was assessed by examining levels of their marker proteins Iba1 and GFAP in the cortex tissues harvested from mice sacrificed 48 h post-WD-TBI (Figure 4). Western blot data revealed marked upregulation in Iba1 and GFAP levels at 48 h in the WD group compared with the Control group (Figures 4A,B), demonstrating WD-TBI-associated activation of microglia and astrocytes. Importantly, elevation of these neuroinflammatory proteins was inhibited by EA treatment (WD group vs. WD+EA group, *p <* 0.05, Figure 4B). IHC analysis was also performed to evaluate Iba1 and GFAP levels in the Control, WD, WD+EA, and WD+EA-NAP groups (Figures 4C–F). Representative images of mouse cortical and hippocampal areas in the Control group revealed small, weakly-stained Iba1-positive cell bodies with several thin processes (Figures 4C,D), with the appearance of “resting” or “ramified” microglial cells (Ling and Wong, 1993). In contrast, cortex sections from the WD group showed numerous, densely situated, and strongly labeled Iba1-positive cells that appeared hypertrophic, with stout cell processes emanating from the enlarged cell bodies. Tissue from the WD+EA group had weakly-stained Iba1-positive cell bodies with thinner processes, which was like the staining seen in the Control group. Tissue from WD+EA-NAP mice exhibited strongly-stained Iba1-positive cells, similar to those in the WD group tissue. For GFAP staining, representative images of mouse cortical and hippocampal areas from the WD group revealed hypertrophic cell bodies with thick processes in the WD-TBI cortex when compared with tissue from the Control group (Figures 4E,F). In contrast, tissue from the WD+EA group had weakly-stained GFAP-positive cell bodies with thinner processes, similar to the characteristics of the Control group. As expected, tissue from the WD+EA-NAP mice exhibited strongly-stained GFAP-positive cell bodies with thick processes, that resembled staining in the WD group. These results suggest that EA treatment protects against WD-TBI-induced neuroinflammation by reducing microglial and astrocyte activation.

**FIGURE 5:**
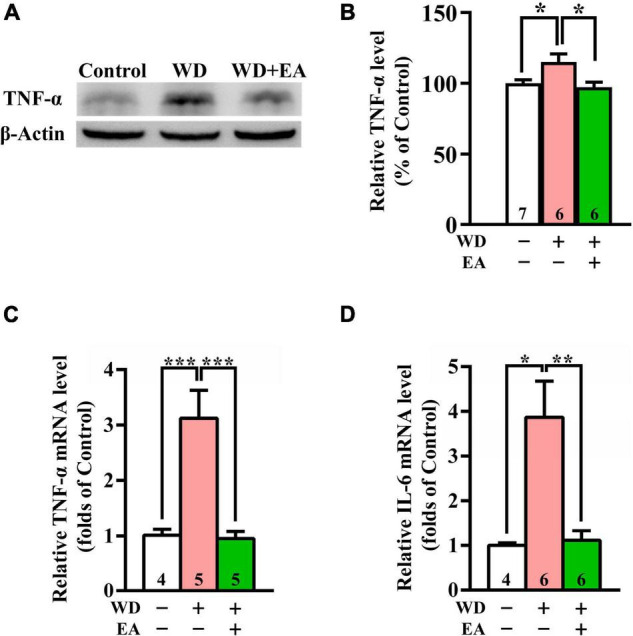
EA treatment reduced TNF-α and IL-6 expression in the WD-TBI mouse cortex. **(A)** Representative Western blot results show levels of TNF-α expression in the cortex from the Control, WD, and WD+EA groups 48 h after TBI. β-Actin was used as the loading control. **(B)** Quantitative analysis of TNF-α protein expression levels in **(A)**. **(C,D)** The relative mRNA expression levels of TNF-α and IL-6 in the cortex 48 h after TBI were evaluated by qPCR. Data are the means ± S.E.M. of at least three independent experiments. *p < 0.05, **p < 0.01, ***p < 0.001 (statistical testing was performed by one-way ANOVA/Newman-Keuls test). The number underneath each bar in **(B**–**D)** refers to the number of mice used in the study group. The original Western blot images are presented in Supplementary Figure 6. The *F* values of one-way ANOVA for **(B**–**D)** are presented in Supplementary Table 4.

To study the effects of EA on WD-induced neuroinflammation, the expression levels of TNF-α and IL-6 were evaluated using Western blot or qPCR analysis (Figures 5A–D), respectively. The Western blot and qPCR data revealed markedly upregulated expression of TNF-α protein and mRNA levels in the WD group compared with the Control group after 48 h of WD-TBI (*p* < 0.05 in Figure 5B; *p* < 0.001 in Figure 5C). Moreover, mRNA levels of *IL-6* also significantly increased in the WD group compared with the Control group (*p* < 0.05, Figure 5D). Importantly, the elevations of both neuroinflammatory proteins were all inhibited by EA treatment (WD group vs. WD+EA group, *p* < 0.05 in Figure 5B, *p* < 0.001 in Figure 5C, *p* < 0.01 in Figure 5D). The results indicated that WD-TBI upregulated the expression levels of neuroinflammatory TNF-α and IL-6 while EA treatment markedly attenuated these changes induced by WD-TBI.

### Electroacupuncture reduced neuronal apoptosis, activated the Akt and GSK-3β pathways, and upregulated BDNF levels in weight drop-impact acceleration-induced traumatic brain injury mice

In addition to mediating many other neuronal functions, GSK-3β is an important downstream target of the Akt signaling pathway and plays a major role in determining cell fate in the brain. Akt is activated when phosphorylated on serine 473; activated Akt inhibits GSK-3β activity *via* serine 9 phosphorylation (Hu et al., 2013); this action is essential for cell survival (Häggblad Sahlberg et al., 2017).

**FIGURE 6:**
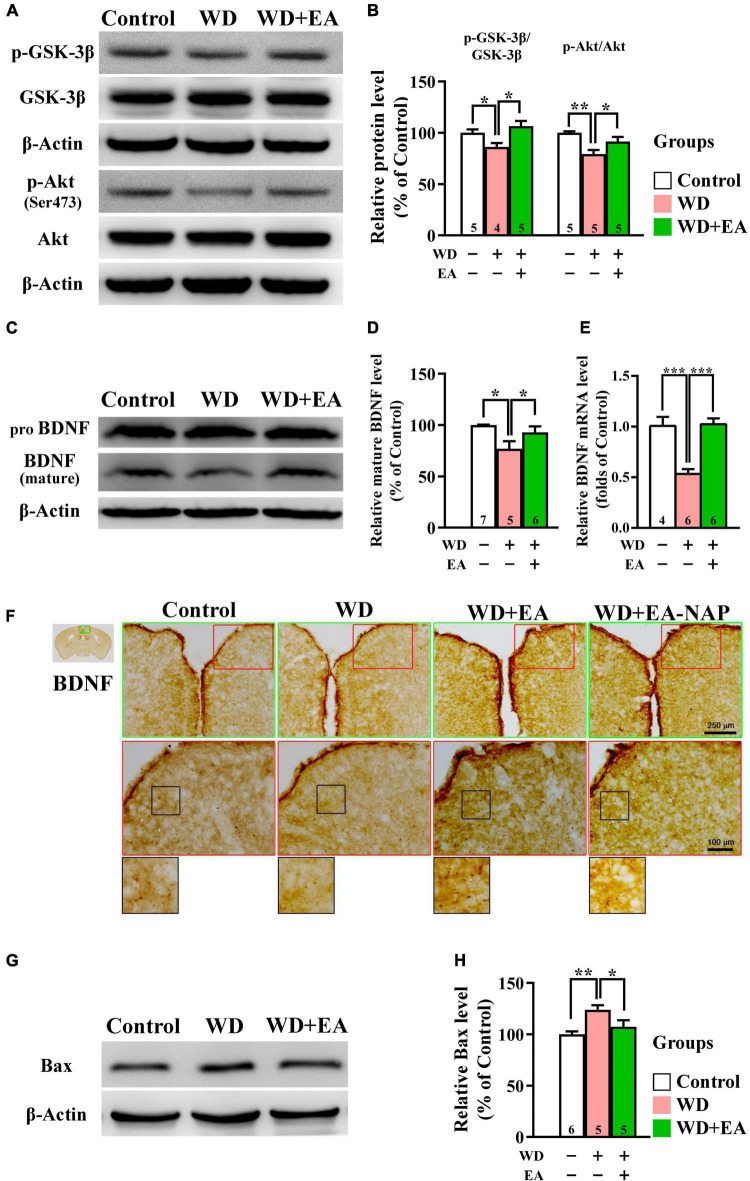
EA treatment restored downregulation of neuroprotective p-GSK-3β, p-Akt, BDNF, and Bax in the WD-TBI mouse cortex. **(A,C)** Representative Western blot results show levels of p-GSK-3β, p-Akt **(A)**, and mature BDNF **(C)** in the mouse cortex from the Control, WD, and WD+EA groups. β-Actin was used as the loading control. **(B,D)** Quantitative analysis of p-GSK-3β, p-Akt, and mature BDNF protein expression levels in **(A,C)**, respectively. **(E)** The relative mRNA expression levels of BDNF in the cortex 48 h after TBI were evaluated by qPCR. **(F)** Representative images of IHC staining show anti-BDNF antibody immunoreactivity in cortex samples in the Control, WD, WD+EA, and WD+EA-NAP groups. High magnification images marked by the small red boxes from the top panels (green box) are shown below (red box). The bottom panels in **(F)** show higher-magnification images of the cortex from the middle panels marked by the small black boxes. Scale bars of the green box panels are 250 μm; of the red box panels are 100 μm. **(G)** Representative Western blot results show levels of Bax expression in cortex from the Control, WD, and WD+EA groups 48 h after TBI. β-Actin was used as the loading control. **(H)** Quantitative analysis of Bax protein expression levels in **(G)**. Data are the means ± S.E.M. of at least three independent experiments. *p < 0.05, **p < 0.01, ***p < 0.001 (statistical testing was performed by one-way ANOVA/Newman-Keuls test). The number underneath each bar in **(B)**, **(D)**, **(E)**, and **(H)** refers to the number of mice used in the study group. The original Western blot images are presented in Supplementary Figures 7–9. The *F* values of one-way ANOVA for **(B)**, **(D)**, **(E)**, and **(H)** are presented in Supplementary Table 5.

To investigate the effects of EA treatment on the Akt/GSK-3β signaling pathway following WD-TBI, Western blot assays were performed to assess cortical levels of p-GSK-3β and upstream BDNF expression, as well as p-Akt and downstream pro-apoptotic Bcl-2-associated X protein (Bax) at 48 h after WD-TBI. In the WD group, Western blotting and quantitative analysis revealed significant decreases in cortical p-GSK-3β and p-Akt levels compared with the Control group, but no changes in total protein levels (Figures 6A,B), suggesting GSK-3β activation and Akt inhibition after WD-TBI. Notably, EA treatment largely prevented these WD-TBI-induced losses of p-GSK3β and p-Akt in the cortex of the WD+EA group (WD group vs. WD+EA group, *p <* 0.05, Figure 6B). Protein levels of BDNF, which is the upstream regulator of Akt/GSK-3β and whose expression is reciprocally regulated by GSK-3β and HDACs (including HDAC1) (Yasuda et al., 2009), were subsequently investigated. Cortical levels of mature BDNF were significantly lower in the WD group 48 h post-TBI compared with the Control group (*p <* 0.05, Figures 6C,D); this WD-induced BDNF downregulation was restored by EA treatment (WD group vs. WD+EA group, *p <* 0.05). Moreover, similar trends of BDNF expression were reconfirmed at the mRNA level in the WD group compared with the WD+EA group (*p <* 0.001, Figure 6E).

IHC staining images revealed weak BDNF staining in the cortices of both the Control and WD groups, whereas BDNF staining was stronger in the cortices of mice in the WD+EA group (Figure 6F). Changes in protein levels of the apoptotic marker Bax were then assessed to determine the anti-apoptotic effects of EA in WD-TBI mice. Cortical Bax levels were significantly increased in the WD group compared with the Control group (*p <* 0.01), and this upregulation was significantly suppressed in WD+EA mice compared to the WD group (*p <* 0.05, Figures 6G,H).

### Electroacupuncture improved functional outcomes in controlled cortical impact-induced traumatic brain injury mice

**FIGURE 7:**
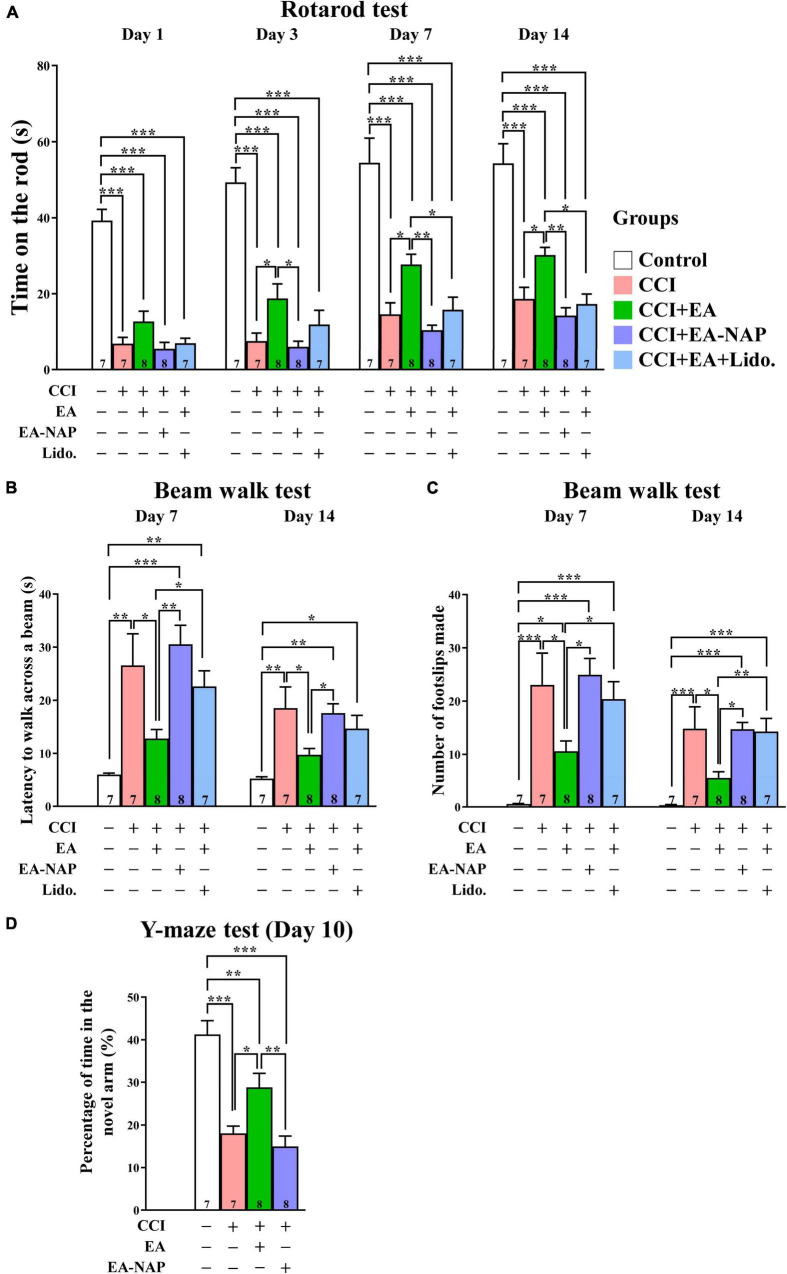
EA treatment improved functional deficits in CCI-TBI mice. **(A)** Length of time spent on the accelerating rotarod on Days 1, 3, 7, and 14. **(B)** Time taken to complete the beam walk and **(C)** number of the beam walk footslips on Days 7 and 14. **(D)** Y-maze testing on Day 10. For the lidocaine-induced radial nerve block, a single injection of lidocaine (2%, 10 μL) was administered at acupoint LI11 before EA. Data are the means ± S.E.M. of at least three independent experiments. *p < 0.05, **p < 0.01, ***p < 0.001 (statistical testing was performed by one-way ANOVA/Newman-Keuls test). The number underneath each bar in **(A**–**D)** refers to the number of mice used in the study group. The detailed data points are presented in Supplementary Figure 10. The *F* values of one-way ANOVA for **(A–D)** are presented in Supplementary Table 6.

To confirm the key results obtained using the WD-TBI model, the studies were repeated using CCI-TBI mice. Functional outcomes were evaluated using the accelerating rotarod, beam walk, and Y-maze tests. In the accelerating rotarod test, the mice in CCI group spent dramatically less time on the rotarod compared with the Control group on Days 1, 3, 7, and 14 (*p <* 0.001 in all cases, Figure 7A). EA treatment improved CCI-TBI-induced endurance deficits on Days 3, 7, and 14 (CCI group vs. CCI+EA group, *p <* 0.05 in all cases). However, treatment at non-acupoints did not improve rotarod performance in CCI+EA-NAP group compared with the CCI group (*p* > 0.05 in all cases). In addition, lidocaine (2%, Lido.) injection at LI11 blocked EA-induced improvements in rotarod performance on Days 7 and 14 (CCI+EA group vs. CCI+EA+Lido. group, both *p <* 0.05), suggesting that EA-induced improvements involved radial nerve stimulation.

Mice in the CCI group were too weak to perform the beam walk test before Day 7, so testing was conducted on Days 7 and 14 after CCI-TBI (Figure 7B). Mice in the CCI group took much longer to complete the beam walk test compared with the Control group on Days 7 and 14 (both *p <* 0.01). The CCI+EA group spent markedly shorter times’ on the beam compared with the CCI group mice on Days 7 and 14 (both *p <* 0.05); no such improvement was noted in the CCI+EA-NAP group compared with the CCI group. Moreover, lidocaine (2%) injection at LI11 blocked EA-induced improvements in beam walk performance on Day 7 (CCI+EA group vs. CCI+EA+Lido. group, *p* < 0.05). On testing Days 7 and 14, significantly more foot faults occurred in the CCI group compared with the Control group (both *p <* 0.001, Figure 7C), but the CCI+EA group had a significantly reduced numbers of foot faults compared with the other CCI-TBI groups (*p <* 0.05 vs. other CCI-TBI groups on Day 7, *p <* 0.01 vs. CCI+EA+Lido. group, *p <* 0.05 vs. other CCI-TBI groups on Day 14). CCI+EA-NAP did not change the number of foot faults induced by CCI-TBI. Moreover, 2% lidocaine injection at LI11 also blocked the effects of EA on both testing days (CCI+EA group vs. CCI+EA+Lido. group, *p* < 0.05 on Day 7, *p* < 0.01 on Day 14). These results suggest that EA is associated with persistent improvement in neurological function. The Y-maze test, which is frequently used to evaluate hippocampal-dependent learning and memory (Yau et al., 2007), was performed on Day 10 (Figure 7D). The CCI group spent more than 50% less time in the novel arm than the Control group, indicating impaired spatial learning and memory in CCI-TBI mice. In contrast, the CCI+EA group spent significantly longer exploring the novel arm than the CCI group (*p <* 0.05). No significant differences in time spent in the novel arm were seen between the CCI+EA-NAP group vs. the CCI group.

### Electroacupuncture lowered levels of GFAP, histone deacetylase 1, and histone deacetylase 3 in controlled cortical impact-induced traumatic brain injury mice

**FIGURE 8:**
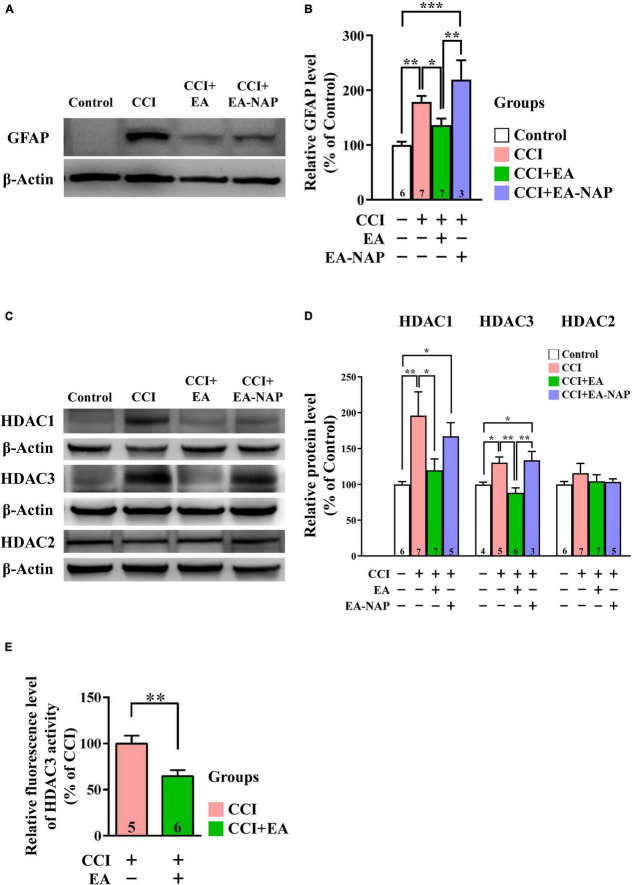
EA lowered GFAP and HDAC1/3 expression levels after CCI-TBI. **(A,C)** Representative Western blot results show protein levels of GFAP **(A)**; HDAC1, HDAC3, and HDAC2 **(C)** in the cortex at 48 h from the Control, CCI, CCI+EA, and CCI+EA-NAP groups. **(B,D)** Quantitative analysis of GFAP **(B)**, HDAC1/3/2 **(D)** protein levels at 48 h in cortical samples from the Control, CCI, CCI+EA, and CCI+EA-NAP groups. Quantitative analysis of GFAP **(B)** and HDAC1/3/2 **(D)** protein expression levels in **(A,C)**, respectively. **(E)** HDAC3 activity in the CCI+EA group was measured and compared with the CCI group, as described in the “Materials and methods.” Cortical tissues were harvested from ≥ 5 mice per group 48 h after CCI injury (or anesthesia only, in the controls). Data are the means ± S.E.M. of at least three independent experiments. *p < 0.05, **p < 0.01, ***p < 0.001 [Statistical testing was performed by one-way ANOVA with Newman-Keuls test in **(B,D)** and by Student’s *t*-test in **(E)**]. The number underneath each bar in **(B,D,E)** refers to the number of mice used in the study group. The representative images were cropped from the original Western blot images, which are presented in Supplementary Figures 11, 12. The detailed data points of **(E)** are presented in Supplementary Figure 13. The *F* values of one-way ANOVA for **(B,D)** are presented in Supplementary Table 7.

In Western blotting and quantitative analyses, GFAP levels were markedly upregulated in mice in the CCI group vs. the Control group 48 h after CCI-TBI (*p <* 0.01, Figures 8A,B). GFAP levels were downregulated in the CCI+EA group compared with the CCI group (*p <* 0.05). In addition, GFAP levels in the CCI+EA-NAP group were significantly higher than in the CCI+EA group (*p <* 0.01, Figure 8B). These results indicate that EA, but not EA-NAP, suppressed CCI-TBI-induced GFAP-associated inflammation. Similar to findings in the WD-TBI model, HDAC1 and HDAC3 protein levels were markedly upregulated in the cortex 48 h after CCI-TBI in the CCI group compared with the Control group (*p <* 0.01 in HDAC1, *p <* 0.05 in HDAC3, Figures 8C,D). This increase was significantly downregulated in the CCI+EA group (CCI group vs. CCI+EA group, *p <* 0.05 in HDAC1, *p <* 0.01 in HDAC3), but no such differences were observed in the CCI+EA-NAP group (Figure 8D). As with the WD group, HDAC2 protein levels did not significantly differ between groups (Figure 8D). When the enzymatic activity of HDAC3 was analyzed using the HDAC3 Activity Fluorometric Assay Kit, EA treatment was found to significantly suppress HDAC3 activity in the CCI+EA group compared with the CCI group (*p <* 0.01, Figure 8E). These data suggest that EA-induced neuroprotection in CCI-TBI is associated with enzymatic inhibition of certain HDAC isoforms.

## Discussion

In the present study, we used the WD- and CCI-TBI models to assess the effects of EA treatment. These two commonly used animal models of TBI are designed to mimic the clinical consequences of injury mechanisms resembling human TBI. In both models, the major pathological features are contusion, hemorrhage, concussion, and traumatic axonal injury (Xiong et al., 2013). The CCI-TBI model is associated with widespread neurodegeneration in the cortex, hippocampus, and thalamus, providing anatomical correlates for the cognitive and motor dysfunction observed after penetrative brain injury (Hall et al., 2005). WD-TBI injuries are characterized by widespread, bilateral damage in the neurons, axons, dendrites, and microvasculature. In addition, WD induces extensive diffuse axonal injury in the corpus callosum, internal capsule, optic tracts, cerebral and cerebellar peduncles, as well as the long tracts in the brainstem (Xiong et al., 2013). These injuries mimic human diffuse TBI caused by falls or motor vehicle accidents (Xiong et al., 2013; Giza et al., 2018).

This study identified significant beneficial outcomes for EA treatment after experimental TBI, with confirmatory results from both WD- and CCI-TBI mouse models. Consistent with previous work (Xiong et al., 2013), severe deficits in motor, coordination, and sensorimotor functioning were observed in both TBI models, and EA treatment at acupoints L14 and LI11 robustly improved motor coordination and sensorimotor deficits; notably, sham acupuncture produced no such benefits. Hippocampus-dependent learning/memory was also severely impaired in CCI-TBI mice, a deficit that was similarly significantly alleviated by EA treatment at verum acupoints, but not at non-acupoints. Furthermore, all these EA-induced behavioral improvements were blocked by lidocaine injection (a local anesthetic), suggesting that radial nerve stimulation is required for EA-induced neuroprotective effects. Another salient finding was that EA at LI4 and LI11 stimulated the radial nerve and then downregulated or inhibited HDAC1 and HDAC3 activity in the brain cortex to elicit neuroprotective effects via BDNF-associated Akt/GSK-3β signaling. To the best of our knowledge, this is the first report showing that TBI induces overexpression of certain HDAC isoforms in the brain and that EA treatment can normalize their overexpression. Furthermore, this study robustly demonstrates for the first time that EA treatment is beneficial in a variety of TBI-induced deficits in motor, coordination, sensorimotor functioning, and learning/memory.

A significant advantage of working with both the WD- and CCI-TBI mouse models include their clinical relevance; the WD reproduces predominantly diffuse brain injury and closely recapitulates clinical presentations of cerebral contusion, while the CCI generates focal brain injury and demonstrates an excellent correlation between the magnitude of cortical deformation and the histological damage sustained by the brain (Zhang et al., 2014). The results add to a growing body of literature showing that acupuncture elicits neuroprotective effects in the CNS and improves motor function after ischemic stroke (Fleminger et al., 2003; Bai et al., 2014), improves gait performance in patients with Parkinson’s disease (Fukuda and Egawa, 2015), and increases serum BDNF levels and reduces Hamilton Depression Rating Scale scores in patients with Parkinson’s disease and depression (Xia et al., 2012).

It is generally accepted that neuroinflammation resulting from the activation of microglia and astrocytes plays a central role in mediating the pathophysiology of TBI. After brain injury, the resulting overexpression of inflammatory cytokines such as TNF-α induces an inflammatory response and subsequent neurodegeneration. As expected, this study found that levels of the microglial marker Iba1, the astrocyte marker GFAP, and the inflammatory cytokine TNF-α were significantly enhanced in the mouse cortex 48 h after TBI, as revealed by Western blotting and IHC assays. EA treatment at LI4 and LI11 acupoints attenuated all these neuroinflammatory responses and mitigated HDAC3 enzymatic activity at 48 h after CCI injury. Our assessment of neurobiochemical events underlying EA-induced behavioral improvements focused on the effects of EA on HDAC expression and BDNF-associated Akt/GSK-3β signaling, both of which are involved in regulating inflammation and apoptosis. Notably, cortical protein levels of HDAC1 and HDAC3, but not HDAC2, were significantly increased after TBI, and this upregulation was abolished by EA treatment in both TBI models. HDAC upregulation after TBI would be expected to modify histone acetylation and alter gene transcription, resulting in the expression of proapoptotic and proinflammatory molecules. Given that pan-histone deacetylase inhibitors exhibit beneficial effects against acute brain injury by suppressing injury-induced neuroinflammation, apoptosis/brain lesions, and neurological deficits (Kim et al., 2007; Chuang et al., 2009; Gibson and Murphy, 2010; Shukla and Tekwani, 2020), our novel finding of EA-induced inhibition of the class I HDAC isoforms HDAC1 and HDAC3 may have strong therapeutic implications. Interestingly, short interfering RNA silencing of HDAC1 in primary cortical neurons activates *BDNF* gene expression by enhancing its promoter IV activity (Yasuda et al., 2009), while HDAC3 silencing is responsible in part for elongating astrocytic processes in cultures treated with the HDAC inhibitor valproate (Leng et al., 2016). In this study, HDAC2 levels in cortical tissues were unchanged after TBI and unaffected by EA treatment in both TBI models. Conversely, it was recently reported that levels of HDAC2 were upregulated in the spinal cord after TBI, with an accompanying downregulation in spinal BDNF (Sada et al., 2020). These results suggest that different class I HDAC isoforms have distinct modulatory and functional roles in the CNS after injury.

The BDNF-associated PI3K/Akt/GSK-3β axis is one of the most prominent pathways in determining the life and death of neurons and neutrally-related cells (Hu et al., 2013; Matsuda et al., 2019). GSK-3 is a master-switch protein kinase involved in a diverse array of cellular functions, including neuroprotection. Thus, the GSK-3-associated mechanism of neuroprotection is not restricted by neurons only; other cell types of the neurovascular unit are also involved and could serve as targets for neuroprotection as well (Leeds et al., 2014; Bosche et al., 2016; Shim and Stutzmann, 2016; Ciftci et al., 2020; Haupt et al., 2020). The constitutively active forms, GSK-3α and GSK-3β, are considered to be proapoptotic, while phosphorylation of GSK-3α or GSK-3β by Akt (one of the upstream kinases) inhibits GSK-3 activity and confers neuroprotective properties (Chiu and Chuang, 2010). Akt plays a central role in cell survival signaling and is activated when phosphorylated at Ser473 by upstream BDNF-associated TrkB/PI3K signaling (Hu et al., 2013; Matsuda et al., 2019). The results demonstrate that TBI markedly downregulated p-GSK-3β at Ser29 and p-Akt at Ser473 and decreased BDNF protein levels and immunoreactivity in the brain cortex 48 h after TBI. Importantly, EA at LI4 and LI11 acupoints blocked all these TBI-induced decreases, strongly suggesting that the BDNF-associated Akt/GSK-3β signaling pathway is a critical target for EA to elicit beneficial effects in TBI mice. GSK-3 is also increasingly believed to be a key regulator of innate and adaptive immunity, and GSK-3 inhibition is thought to reduce proinflammatory cytokine production, but increase anti-inflammatory cytokine production by microglia within the CNS (Beurel, 2011). In the CCI-TBI mouse model, post-insult treatment with lithium, a well-known GSK-3 inhibitor, profoundly alleviated microglial activation and cyclooxygenase-2 (COX-2) overexpression in neurons, concomitant with increased p-GSK-3β expression and subsequent behavioral improvement (Yu et al., 2012). Molecular mechanisms underlying anti-inflammatory effects induced by inhibition of GSK-3 or HDACs may involve superinduction of heat shock protein 70, inactivating the proinflammatory protein-associated nuclear factor kappa B (NF-κB) by stabilizing the NF-κB-IκB complex (Ren et al., 2003; Yenari et al., 2005; Kim et al., 2007; Wang et al., 2011).

Apoptosis is one of the cellular events that occurs during secondary injury post-TBI and is characterized by induction of proapoptotic proteins, including p53 and Bax (Wennersten et al., 2003; Rana et al., 2019). This study demonstrated that cortical Bax protein was overexpressed post-TBI and, furthermore, that this overexpression was essentially blocked by EA treatment. Bax mediates the release of proapoptotic proteins, including cytochrome *c* from the mitochondria, facilitating caspase activation in the cytosol (Zhang et al., 2017). Lithium-induced blockade of Bax and p53 upregulation during excitotoxicity in primary neurons (Chen and Chuang, 1999) and valproate-mediated blockade of p53 upregulation after cerebral ischemia (Kim et al., 2007) are most likely mediated by inhibition of GSK-3 and HDACs, respectively. Indeed, HDAC inhibitors suppress the transcription of p53 and Bax in mouse brain (Wang et al., 2019) and block both p53- and Bax-dependent neuronal apoptosis, as well as p53-independent but Bax-dependent neuronal apoptosis (Uo et al., 2009).

**FIGURE 9:**
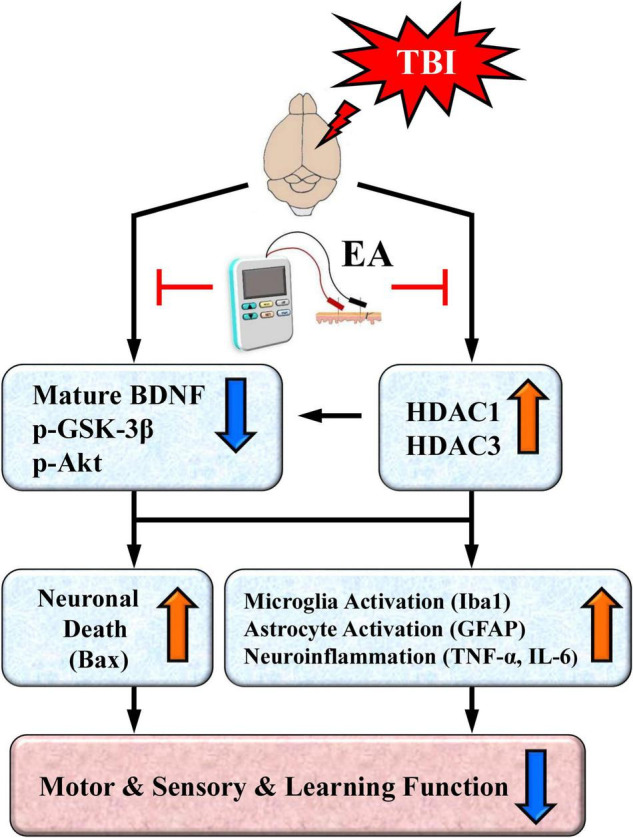
A proposed working model depicting neurochemical, neurobiological, and behavioral changes after traumatic brain injury (TBI) and their reversal or protection by EA after the insult. TBI induces overexpression of certain HDAC isoforms, notably HDAC1 and HDAC3. The cell survival BDNF-associated Akt/GSK-3β signaling pathway is also downregulated after TBI; this could be directly mediated by the brain injury or indirectly mediated through overexpressed HDACs. Either the overexpression of HDACs or aberrant BDNF-associated Akt-GSK-3β signaling may lead to activation of microglia and astrocytes as well as increased expression of the proinflammatory cytokine TNF-α and IL-6. Either of these aberrant neurochemical events may also trigger overexpression of the proapoptotic factor Bax to induce neuronal apoptosis. The end result is marked deficits in motor and sensory behavioral performance as well as impaired learning and memory. EA treatment at the acupoints normalizes these neurobiological events and robustly facilitates behavioral recovery.

Based on existing results and our recent findings, we propose a working model that depicts the key neurobiochemical and neurobehavioral changes after TBI and their reversal or protection by EA treatment (Figure 9). As illustrated in the working model, TBI induces overexpression of certain HDAC isoforms, notably HDAC1 and HDAC3, and thus enhances their deacetylase activity. Neuroprotective BDNF-associated Akt/GSK-3β signaling is also downregulated after TBI. HDAC overexpression and aberrant BDNF-associated Akt/GSK-3β signaling, alone or in combination, may activate microglia and astrocytes, as well as increase proinflammatory cytokine TNF-α expression. These neurobiochemical events may also trigger overexpression of the proapoptotic factor Bax, resulting in neuronal cell apoptosis. These TBI-induced events ultimately produce marked deficits in sensorimotor performance as well as impaired learning and memory.

Our results here indicate that EA at the LI4 and LI11 acupoints normalized neurobiochemical and neurobiological events, and facilitated functional recovery after TBI. However, it remains unknown whether TBI-induced downregulation of BDNF-associated Akt/GSK-3β signaling was directly mediated by brain injury or indirectly mediated via HDAC overexpression. The latter possibility is supported by the finding that HDAC1 silencing or HDAC inhibition can upregulate BDNF levels in cultured rat cortical neurons and in the hippocampus of ischemic rats (Kim et al., 2009; Yasuda et al., 2009).

There are several limitations of the present study. Gender may affect many aspects of the TBI pathological features, such as clinical manifestations and cognitive impairments (Ma et al., 2019). Previous studies demonstrated that female animals exert improved behavioral outcomes and pathophysiological changes following TBI, compared to male animals (Ma et al., 2019; Rubin and Lipton, 2019). It is known that females respond differently to brain injury compared with males, possibly due to the influence of female hormones. However, the specific mechanisms underlying these gender differences remain unclear. Our study is limited to studying the neuroprotective effects of EA only in aged-matched male mice following TBI by using different behavioral and biochemical techniques.

According to the characteristics of the WD- and CCI-TBI models mentioned before, we chose the Y-maze test to evaluate reference and working memory, as well as spatial learning in rodents in the early stage of TBI, and found that EA is beneficial to the learning/memory deficits caused by TBI in the Y-maze test. We have also performed the rotarod test, beam walk test, and adhesive removal test to confirm the motor-sensory benefits of EA. The rotarod test has been reported to be the most efficient and reliable test for measuring the functional recovery of the TBI model (Fujimoto et al., 2004; Zhang et al., 2022). The beam walking test was recognized to be an effective and sensitive method of assessing fine coordination and balance (Luong et al., 2011; Zhang et al., 2022). Moreover, the adhesive removal test was established in rats to determine the long-term effects of brain injury on sensory-motor behavior. It evaluates sensory-motor impairments after unilateral lesions involving the sensory-motor cortex, corticospinal tract, and striatum (Balkaya et al., 2013). The effects of EA on additional TBI-induced behavioral deficits assessed by other functional assessments require further studies. It is also unknown whether the EA-induced biochemical events and behavioral benefits observed in our studies using animal TBI models can be translated into clinical settings of TBI. Given that EA treatment is safe and essentially non-invasive and produces little side effects, timely clinical trials using EA for TBI victims seem to be well justified.

Previous studies demonstrated beneficial effects of acupuncture treatment following ischemic stroke, which shares some pathophysiological features with TBI (Feng and Zhang, 2014). Potential mechanisms underlying protection against stroke-induced brain injury and behavioral deficits may include promotion of cell proliferation and neurogenesis in the brain, improvement of cerebral blood flow, suppression of apoptosis and brain infarction, as well as enhancement of long-term potential and synaptic activity (Chavez et al., 2017). Future investigation will be required to assess the roles of these events in mediating the effects of EA in our TBI experimental conditions.

Furthermore, previous research has demonstrated that treatment of TBI mice with valproate and/or lithium significantly attenuated TBI-induced blood-brain barrier (BBB) disruption (Yu et al., 2012, 2013). It is certainly of interest and importance to assess whether EA’s beneficial effects in TBI mice involve protection against TBI-induced BBB disruption. Future studies are needed to address this question.

To date, no pharmacological therapies have been shown to cure moderate-to-severe TBI, nor have any pharmacotherapies been shown to unequivocally improve functional outcomes (Khellaf et al., 2019; Crupi et al., 2020; Lerouet et al., 2021). Thus, there is an enormous unmet need for treatments that can effectively improve neurological outcomes and improve quality of life after TBI. The use of EA in TBI offers a treatment option that is effective, safe, and lacks the adverse effects associated with pharmacological therapies. Moreover, one important clinical advantage of EA therapy is that it can be tailored to the individual patient’s specific needs throughout a treatment program, and can thus achieve the best possible results for long-term return of functions (Wolf et al., 2015).

## Conclusion

Our current results indicate that EA is capable of normalizing TBI-induced HDAC overexpression and the aberrant BDNF-associated Akt/GSK-3β pathway, affecting both neuroinflammation and neuronal cell death. This study also provides evidence showing that EA can effectively mitigate several functional behavioral deficits in motor, sensorimotor, and learning/memory. These results advance our knowledge about the efficacy of EA treatment in TBI and warrant exploration in clinical trials.

## Data availability statement

The original contributions presented in this study are included in the article/Supplementary material, further inquiries can be directed to the corresponding author.

## Ethics statement

The animal study was reviewed and approved by Institutional Animal Care and Use Committee of China Medical University, Taiwan (approval number: CMUIACUC-2019-075-1).

## Author contributions

D-MC, ND, and Y-HC conceived the study. S-YH and YH-C designed the research for this study. H-YC, S-TL, Y-TC, and Y-HC performed TBI procedures, behavioral testing and analysis of IHC, and Western blots. S-YC, H-YC, Y-TC, S-TL, and S-YH analyzed the data and generated the figures. PK, S-YH, S-YC, and IM wrote the first draft of the manuscript. D-MC, ND, and YH-C revised the manuscript. D-MC, ND, L-YY, L-LH, and YH-C supervised the study. All authors read and approved the final manuscript.

## Abbreviations

BBB: blood-brain barrier
BDNF: brain-derived neurotrophic factor
CNS: central nervous system
CCI: controlled cortical impact
EA: electroacupuncture
GFAP: glial fibrillary acidic protein
GSK-3: glycogen synthase kinase-3
HDAC: histone deacetylase
IHC: immunohistochemistry
Iba1: ionized calcium-binding adapter molecule 1
PI3K: phosphatidylinositol 3-kinase
p-Akt: phosphorylated-Akt
p-GSK -3 β: phosphorylated-GSK-3 β
TBI: traumatic brain injury
trkb: tropomyosin receptor kinase B
TNF- α: tumor necrosis factor-alpha
WD: weight drop-impact acceleration.
