# Supplementary material for: Electroacupuncture improves TBI dysfunction by targeting HDAC overexpression and BDNF-associated Akt/GSK-3β signaling
Source: Front Cell Neurosci. 2022 Aug 9;16:880267. doi: 10.3389/fncel.2022.880267 (PMC9396337; doi:10.3389/fncel.2022.880267)
Supplement: Supplementary file 1 [file Data_Sheet_1.docx]

**Supplementary Figures S1 – S13**

| (A) | (B) |
| --- | --- |
| **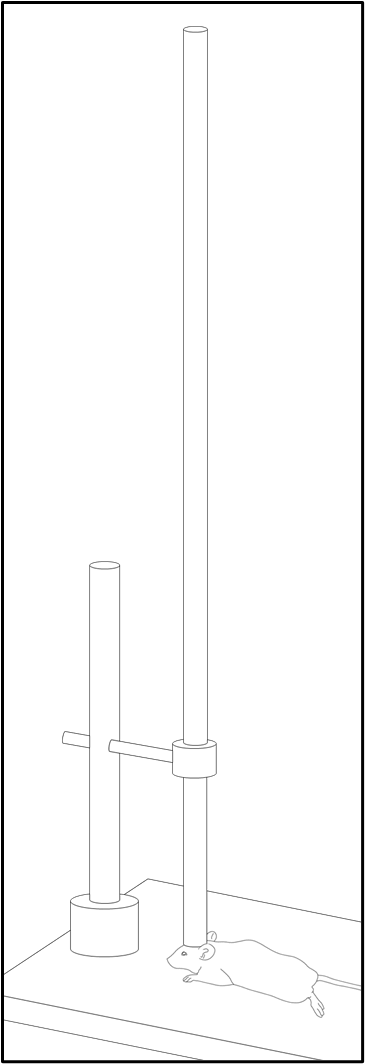** | 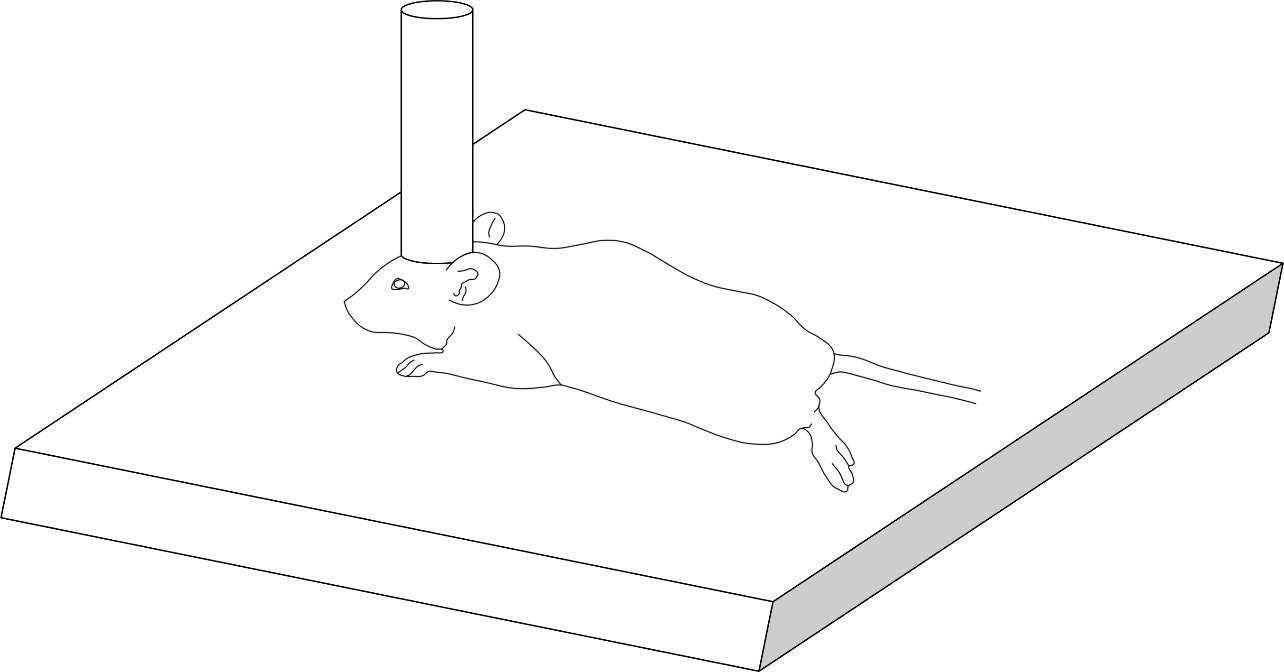  **Supplementary Figure S1.** Illustration of weight drop-impact acceleration model (WD)-induced traumatic brain injury (WD-TBI). (A) The appearance of the device. (B) Application position of impact. |

| (A) | (B) |
| --- | --- |
| 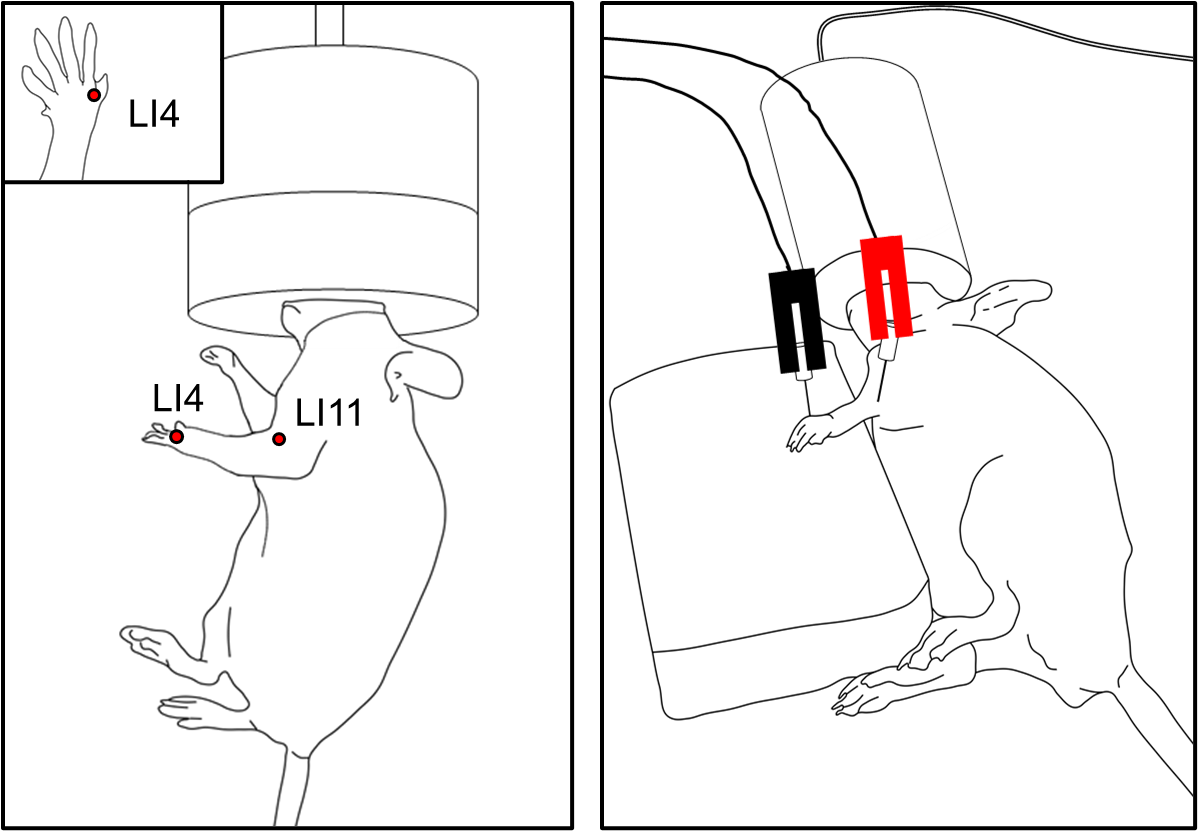 | |

**Supplementary Figure S2.** Illustration of electroacupuncture (EA) treatment. (A) Location of LI4 and LI11 acupoints. (B) Application of EA.


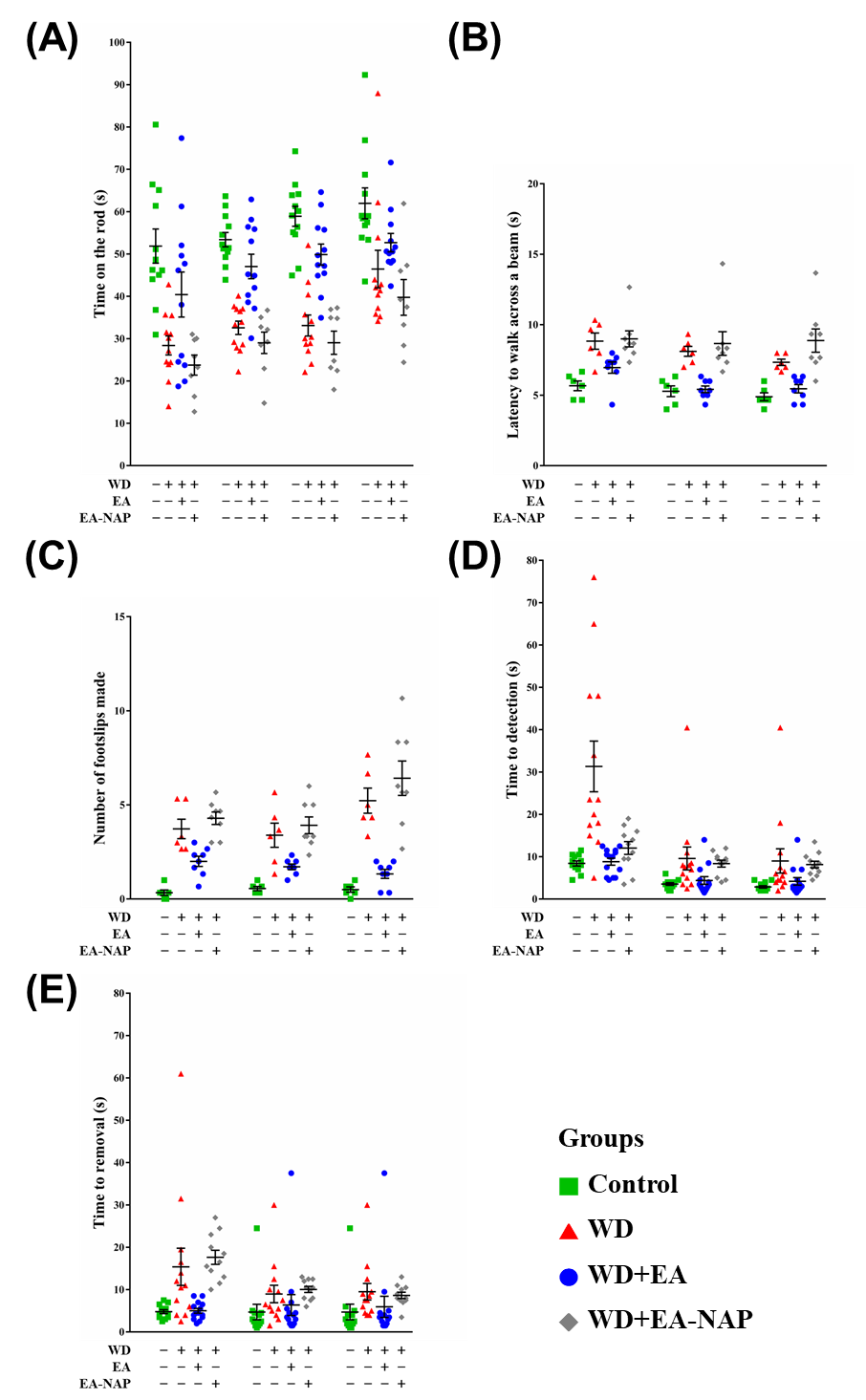


**Supplementary Figure S3.** Individual data points are presented from Figure 2. (A) Fig. 2A. (B) Fig. 2B. (C) Fig. 2C. (D) Fig. 2D. (E) Fig. 2E. Green icon (■)= Control group; red icon (▲) = WD group; blue icon (●) = WD+EA group; grey icon (◆) = WD+EA-NAP group. Abbreviations: WD = Weight drop-impact acceleration model; EA = Electroacupuncture; EA-NAP = EA at non-acupoints.

| (A) | |
| --- | --- |
| HDAC1 (62 kDa) | |
|   **Control**  **WD**  **WD+EA** |  |
| β-Actin (42 kDa) | |
|   **Control**  **WD**  **WD+EA** |  |

| (B) | |
| --- | --- |
| HDAC3 (49 kDa) | |
|   **Control**  **WD**  **WD+EA** |  |
| β-Actin (42 kDa) | |
|   **Control**  **WD**  **WD+EA** |  |

| (C) | |
| --- | --- |
| HDAC2 (60 kDa) | |
|   **Control**  **WD**  **WD+EA** | 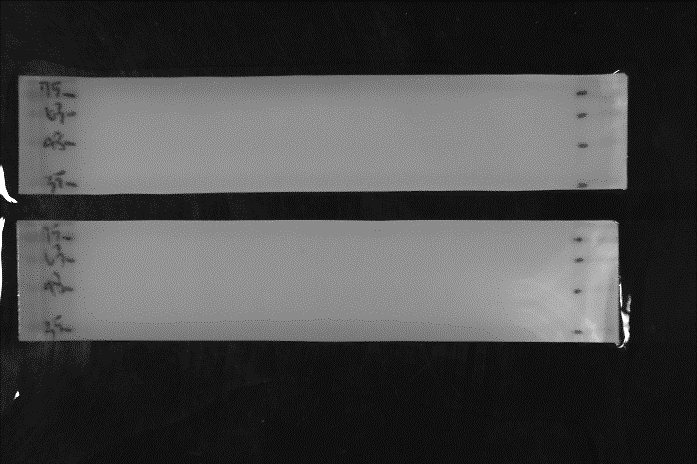 |
| β-Actin (42 kDa) | |
|   **Control**  **WD**  **WD+EA** |  |

**Supplementary Figure S4.** Original Western blot images are presented from Figure 3B. (A) HDAC1. (B) HDAC3. (C) HDAC2. The selected areas indicated by red dashed lines on the bands (Left) are represented by their respective photographs (Right).

| Iba1 (17 kDa) | |
| --- | --- |
|   **Control**  **WD**  **WD+EA** |  |
| GFAP (50 kDa) | |
|   **Control**  **WD**  **WD+EA** |  |
| β-Actin (42 kDa) | |
|   **Control**  **WD**  **WD+EA** | 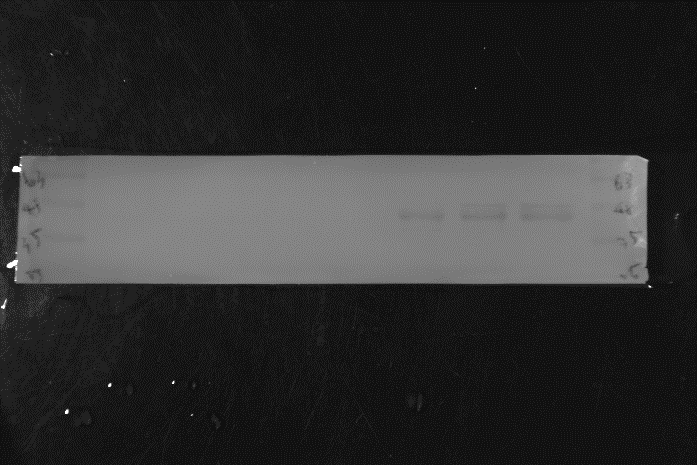 |

**Supplementary Figure S5.** Original Western blot images are presented from Figure 4A. The images of Iba1 (top), GFAP (middle), and β-Actin (bottom). The selected areas indicated by red dashed lines on the bands (Left) are represented by their respective photographs (Right).

| TNF-α (secreted form 17 kDa) | |
| --- | --- |
|   **Control**  **WD**  **WD+EA** | 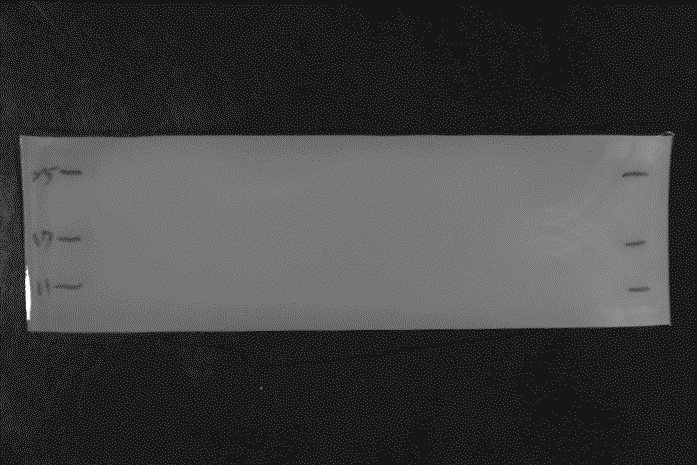 |
| β-Actin (42 kDa) | |
|   **Control**  **WD**  **WD+EA** | 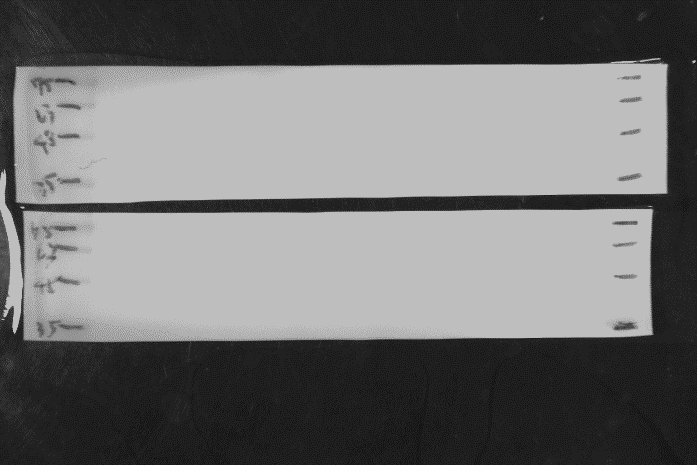 |

**Supplementary Figure S6.** Original Western blot images are presented from Figure 5A. The selected areas indicated by red dashed lines on the bands (Left) are represented by their respective photographs (Right).

| (A) | |
| --- | --- |
| p-GSK-3β (47 kDa) | |
|   **Control**  **WD**  **WD+EA** |  |
| GSK-3β (47 kDa) | |
|   **Control**  **WD**  **WD+EA** |  |
| β-Actin (42 kDa) | |
|   **Control**  **WD**  **WD+EA** |  |

| (B) | |
| --- | --- |
| p-Akt (56 kDa) | |
| 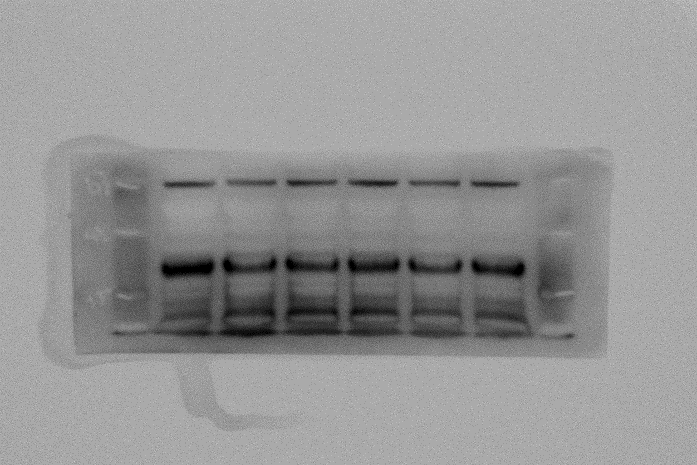  **Control**  **WD**  **WD+EA** |  |
| Akt (56 kDa) | |
| 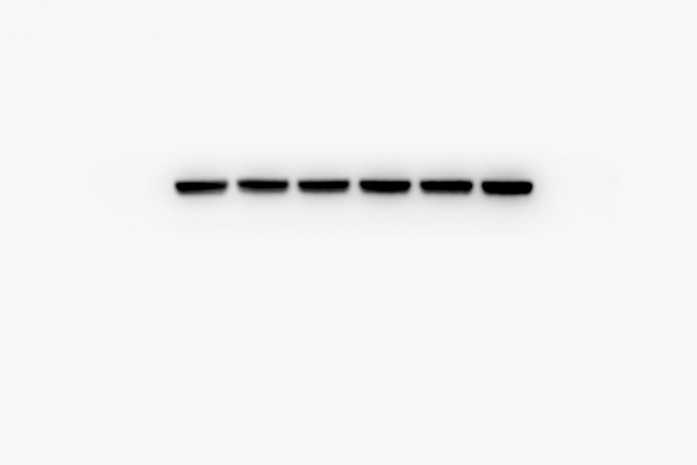  **Control**  **WD**  **WD+EA** |  |
| β-Actin (42 kDa) | |
| 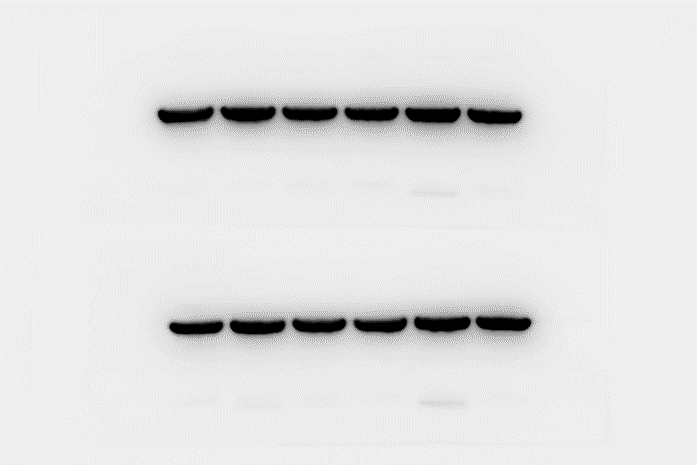  **Control**  **WD**  **WD+EA** |  |

**Supplementary Figure S7.** Original Western blot images are presented from Figure 6A. (A) GSK-3β. (B) Akt. The selected areas indicated by red dashed lines on the bands (Left) are represented by their respective photographs (Right).

| BDNF (14 – 28 kDa) | |
| --- | --- |
|   **pro BDNF**  **BDNF**  **(mature)**  **Control**  **WD**  **WD+EA** |  |
| β-Actin (42 kDa) | |
|   **Control**  **WD**  **WD+EA** |  |

**Supplementary Figure S8.** Original Western blot images are presented from Figure 6C. The selected areas indicated by red dashed lines on the bands (Left) are represented by their respective photographs (Right).

| Bax (20 kDa) | |
| --- | --- |
|   **Control**  **WD**  **WD+EA** |  |
| β-Actin (42 kDa) | |
|   **Control**  **WD**  **WD+EA** |  |

**Supplementary Figure S9.** Original Western blot images are presented from Figure 6G. The selected areas indicated by red dashed lines on the bands (Left) are represented by their respective photographs (Right).


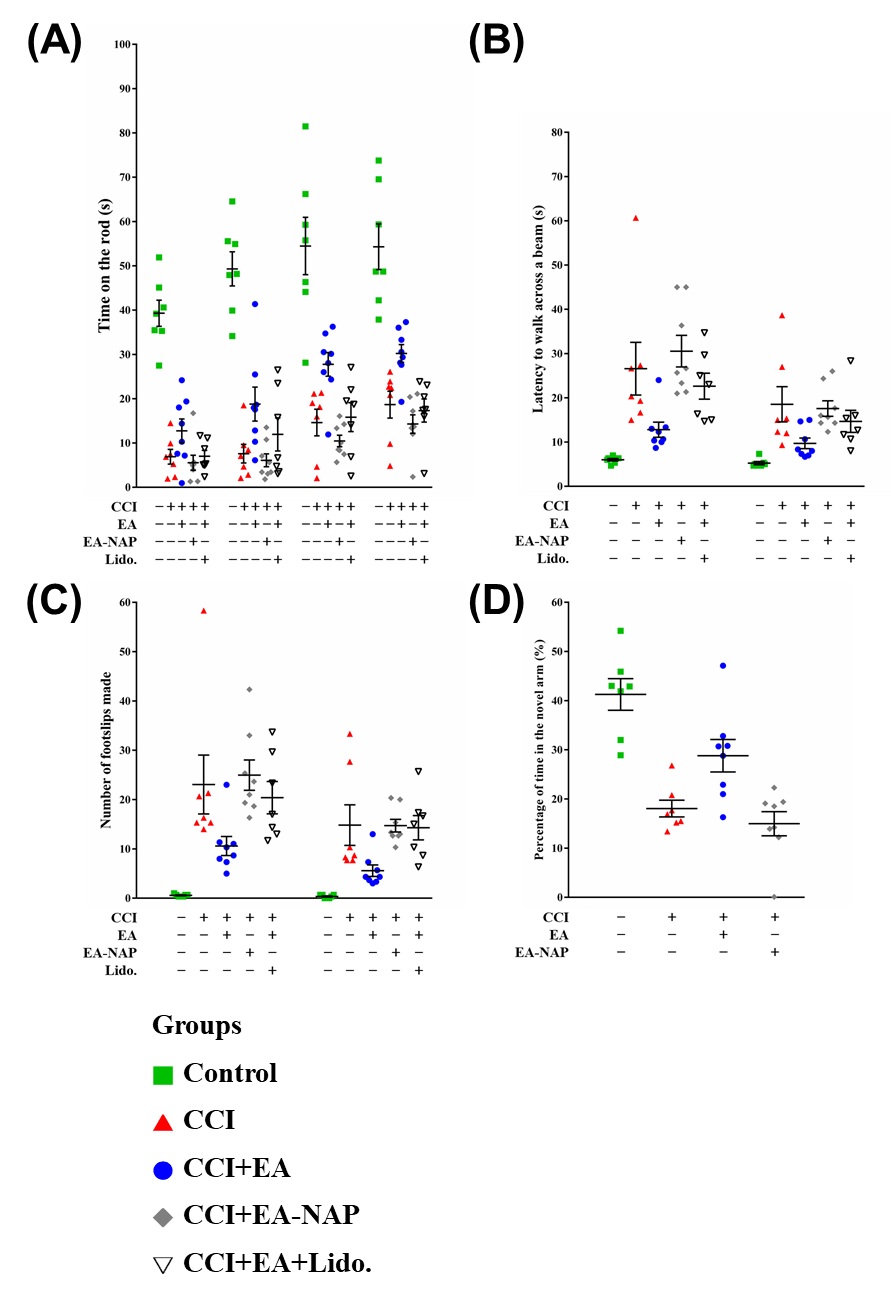


**Supplementary Figure S10.** Individual data points are presented from Figure 7. (A) Fig. 6A. (B) Fig. 6B. (C) Fig. 6C. (D) Fig. 6D. Green icon (■) = Control group; red icon (▲) = CCI group; blue icon (●) = CCI+EA group; grey icon (◆) = CCI+EA-NAP group; hollow icon (▽) = CCI+EA+Lido. group. Abbreviations: CCI = Controlled cortical impact model; EA = Electroacupuncture; EA-NAP = EA at non-acupoints; CCI+EA+Lido. = Lidocaine (2%, 10 μL) injection followed by EA treatment.

| GFAP (50 kDa)  **Control**  **CCI**  **CCI+EA**  **CCI+EA-NAP** | |
| --- | --- |
| 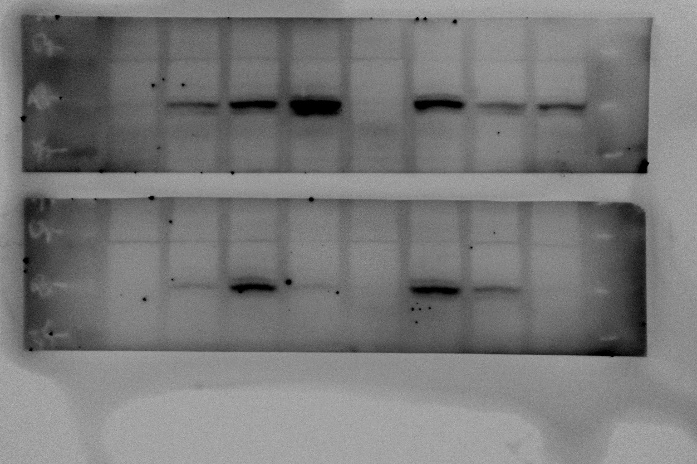 |  |
| β-Actin (42 kDa)  **Control**  **CCI**  **CCI+EA**  **CCI+EA-NAP** | |
| 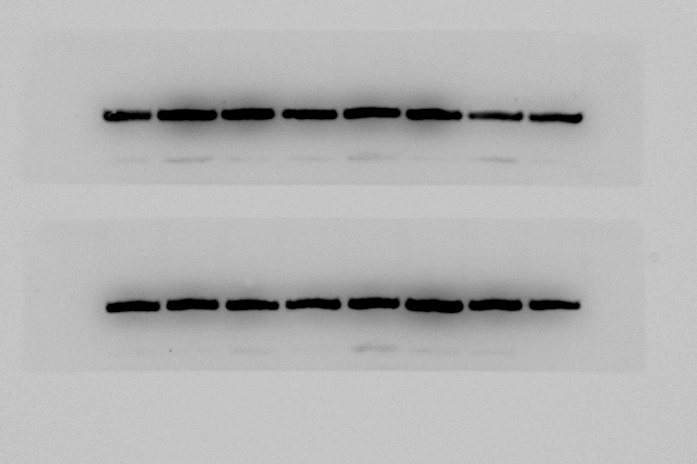 |  |

**Supplementary Figure S11.** Original Western blot images are presented from Figure 8A. The selected areas indicated by red dashed lines on the bands (Left) are represented by their respective photographs (Right).

| (A) | |
| --- | --- |
| HDAC1 (62 kDa) | |
|   **Control**  **CCI**  **CCI+EA**  **CCI+EA-NAP** |  |
| β-Actin (42 kDa) | |
|   **Control**  **CCI**  **CCI+EA**  **CCI+EA-NAP** |  |

| (B) | |
| --- | --- |
| HDAC3 (49 kDa) | |
| 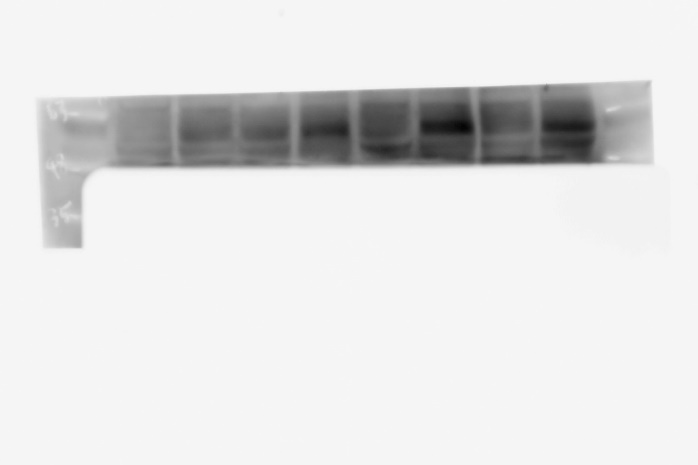  **Control**  **CCI**  **CCI+EA**  **CCI+EA-NAP** |  |
| β-Actin (42 kDa) | |
| 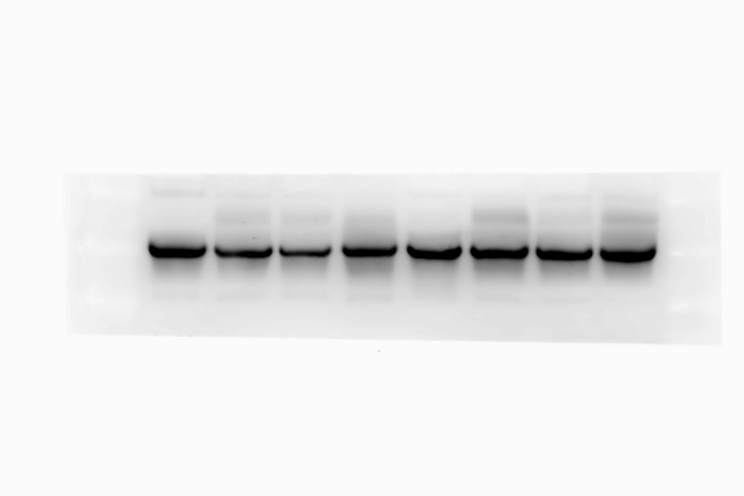  **Control**  **CCI**  **CCI+EA**  **CCI+EA-NAP** |  |

| (C) | |
| --- | --- |
| HDAC2 (60 kDa) | |
|   **Control**  **CCI**  **CCI+EA**  **CCI+EA-NAP** |  |
| β-Actin (42 kDa) | |
|   **Control**  **CCI**  **CCI+EA**  **CCI+EA-NAP** |  |

**Supplementary Figure S12.** Original Western blot images are presented from Figure 8C. (A) HDAC1. (B) HDAC3. (C) HDAC2. The selected areas indicated by red dashed lines on the bands (Left) are represented by their respective photographs (Right).


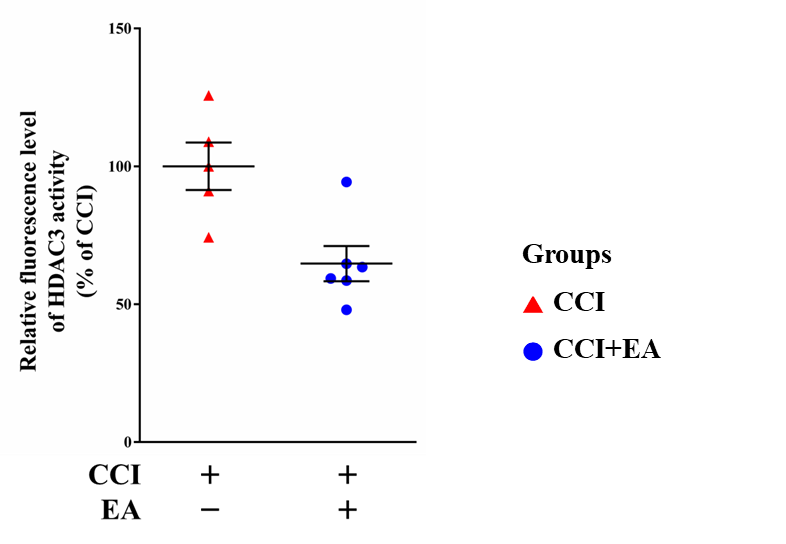


**Supplementary Figure S13.** Individual data points are presented from Figure 8E. Red icon (▲) = CCI group; blue icon (●) = CCI+EA group. Abbreviations: CCI = Controlled cortical impact model; EA = Electroacupuncture.

**Supplementary Tables S1 – S7**

**Supplementary Table S1.**

| **Fig. 2** | **Days** | | | |
| --- | --- | --- | --- | --- |
| **Behavioral testing** | **Day1** | **Day3** | **Day7** | **Day14** |
| **Rotarod test** | F (3, 40) = 9.868  P<0.0001 | F (3, 40) = 26.18  P<0.0001 | F (3, 40) = 30.10  P<0.0001 | F (3, 40) = 6.108  P=0.0016 |
| **Beam walk (time)** | F (3, 24) = 9.959  P=0.0002 | F (3, 24) = 10.61  P=0.0001 | F (3, 24) = 12.30  P<0.0001 |  |
| **Beam walk (foot faults)** | F (3, 24) = 27.17  P<0.0001 | F (3, 24) = 15.53  P<0.0001 | F (3, 24) = 22.31  P<0.0001 |  |
| **Adhesive-detection** | F (3, 46) = 11.75  P<0.0001 | F (3, 46) = 3.632  P=0.0196 | F (3, 46) = 3.374  P=0.0262 |  |
| **Adhesive- remove** | F (3, 46) = 7.645  P=0.0003 | F (3, 46) = 1.422  P=0.2484 | F (3, 46) = 1.291  P=0.2886 |  |

The *F* values of one-way ANOVA in Fig.2 are shown in this table.

**Supplementary Table S2.**

| **Fig. 3** | **Expression levels** |
| --- | --- |
| **Targets** | **Protein** |
| **HDAC1** | F (2, 39) = 4.405  P=0.0188 |
| **HDAC3** | F (2, 33) = 5.313  P=0.0100 |
| **HDAC2** | F (2, 21) = 0.002066  P=0.9979 |

The *F* values of one-way ANOVA in Fig.3 are shown in this table.

**Supplementary Table S3.**

| **Fig. 4** | **Expression levels** |
| --- | --- |
| **Targets** | **Protein** |
| **Iba1** | F (2, 9) = 10.58  P=0.0043 |
| **GFAP** | F (2, 15) = 5.104  P=0.0204 |

The *F* values of one-way ANOVA in Fig.4 are shown in this table.

**Supplementary Table S4.**

| **Fig. 5** | **Expression levels** | |
| --- | --- | --- |
| **Targets** | **Protein** | **mRNA** |
| **TNFα** | F (2, 24) = 5.222  P=0.0131 | F (2, 13) = 20.93  P<0.0001 |
| **IL-6** |  | F (2, 13) = 9.075  P=0.00034 |

The *F* values of one-way ANOVA in Fig.5 are shown in this table.

**Supplementary Table S5.**

| **Fig. 6** | **Expression levels** | |
| --- | --- | --- |
| **Targets** | **Protein** | **mRNA** |
| **p-GSK-3β/ GSK-3β** | F (2, 12) = 6.476  P=0.0124 |  |
| **p-Akt/Akt** | F (2, 15) = 8.746  P=0.0030 |  |
| **BDNF** | F (2, 18) = 4.815  P=0.0211 | F (2, 13) = 22.07  P<0.0001 |
| **Bax** | F (2, 27) = 8.053  P=0.0018 |  |

The *F* values of one-way ANOVA in Fig.6 are shown in this table.

**Supplementary Table S6.**

| **Fig. 7** | **Days** | | | | |
| --- | --- | --- | --- | --- | --- |
| **Behavioral testing** | **Day1** | **Day3** | **Day7** | **Day10** | **Day14** |
| **Rotarod test** | F (4, 32) = 40.92  P<0.0001 | F (4, 32) = 30.77  P<0.0001 | F (4, 32) = 23.80 P<0.0001 |  | F (4, 32) = 27.40 P<0.0001 |
| **Beam walk (time)** |  |  | F (4, 32) = 8.863  P<0.0001 |  | F (4, 32) = 6.131  P=0.0009 |
| **Beam walk (foot faults)** |  |  | F (4, 32) = 9.008  P<0.0001 |  | F (4, 32) = 9.018  P<0.0001 |
| **Y-maze test** |  |  |  | F (3, 26) = 17.94  P<0.0001 |  |

The *F* values of one-way ANOVA in Fig.7 are shown in this table.

**Supplementary Table S7.**

| **Fig. 8** | **Expression levels** |
| --- | --- |
| **Targets** | **Protein** |
| **GFAP** | F (3, 21) = 11.31  P=0.0001 |
| **HDAC1** | F (3, 24) = 4.491  P=0.0123 |
| **HDAC3** | F (3, 16) = 7.200  P=0.0028 |
| **HDAC2** | F (3, 28) = 0.6086  P=0.6150 |

The *F* values of one-way ANOVA in Fig.8 are shown in this table.
